# In pursuit of a better broiler: walking ability and incidence of contact dermatitis in conventional and slower growing strains of broiler chickens

**DOI:** 10.1016/j.psj.2022.101768

**Published:** 2022-01-31

**Authors:** Midian N. Santos, Tina M. Widowski, Elijah G. Kiarie, Michele T. Guerin, A. Michelle Edwards, Stephanie Torrey

**Affiliations:** ⁎ Department of Animal Biosciences, University of Guelph, Guelph, ON, N1G 2W1, Canada; † Campbell Centre for the Study of Animal Welfare, University of Guelph, Guelph, ON, N1G 2W1, Canada; ‡ Department of Population Medicine, University of Guelph, Guelph, ON, N1G 2W1, Canada; § Ontario Agricultural College, University of Guelph, Guelph, ON, N1G 2W1, Canada

**Keywords:** lameness, leg health, growth rate, genotype, slower-growth

## Abstract

In this study, the mobility, incidence, and severity of contact dermatitis and litter moisture content were assessed in 14 strains of broiler chickens differing in growth rate. The strains encompassed 2 conventional (CONV; ADG_0-48_ > 60 g/d) and 12 slower growing (**SG**) strains categorized as FAST (ADG_0-62_ = 53-55 g/d), MOD (ADG_0-62_ = 50-51 g/d), and SLOW (ADG_0-62_ < 50 g/d), with 4 strains in each category. A total of 7,216 mixed-sex birds were equally allocated into 164 pens (44 birds/pen; 30 kg/m^2^) in a randomized incomplete block design, with each strain represented in 8 to 12 pens over 2–3 trials. From each pen, 4 to 6 birds were tested in the latency-to-lie (**LTL**) and group obstacle tests 1 wk prior to the birds reaching 2 target weights (**TWs**) of approximately 2.1 kg (TW1: 34 d for CONV and 48 d for SG strains) and 3.2 kg (TW2: 48 d for CONV and 62 d for SG strains). The incidence of footpad dermatitis (**FPD**) and hock burns (**HB**) were evaluated a day prior to each TW. Litter moisture content was determined biweekly from d 14 to d 56. At TW1, CONV and SLOW had longer LTL than FAST birds. At TW2, CONV, MOD, and FAST birds had similar LTL. At both TWs, CONV birds were lighter than FAST birds in the group obstacle test, yet their number of obstacle crossings was similar. At TW1, CONV birds had greater incidence of FPD than FAST and MOD, while at TW2, CONV birds had greater incidence than the other categories. The incidence of HB in CONV and MOD was greater than SLOW birds at TW1, while at TW2, the incidence of HB was greater in CONV and FAST birds vs. MOD and SLOW birds. Litter moisture content was high in all categories from d 28 onward. Our results indicate that both BW and growth rate influence leg strength and walking ability, whereas the overall high litter moisture content and to a lesser extent growth rate influenced the incidence of contact dermatitis.

## INTRODUCTION

The growing global demand for animal products has contributed to the intensification and growth of the poultry industry (Petracci et al., 2015). Due to improvements in nutrition, health, management strategies, veterinary care, and genetic selection, conventional strains of broiler chickens, commonly referred to as fast-growing (**FG**) strains, have better feed conversion, higher growth rate (> 60 g/d), and reach market weight at an earlier age than ever before (about 2.5 kg in 40 d) (Havenstein et al., 1994, 2003a, b; Doğan et al., 2019; Mancinelli et al., 2020). However, this heavy body weight reached over a short time frame has been linked to the development of bone abnormalities and lameness (Julian, 1998; Bradshaw et al., 2002; Kierończyk et al., 2017).

Lameness is a broad term used to describe impaired walking ability and several debilitating conditions, resulting from multifactorial origins (Sørensen et al., 1999; Bradshaw et al., 2002; Kierończyk et al., 2017). Lame birds commonly show reduced walking ability, difficulty standing, prolonged, frequent squatting while walking, and reduced ability to perform natural and active behaviors the birds may be motivated to perform, leading to frustration (Julian, 1998; McGeown et al., 1999; Weeks et al., 2000; Danbury et al., 2000; Dawkins et al., 2009; Caplen et al., 2013; Vasdal et al., 2018; Norring et al., 2019; Rayner et al., 2020). Lameness can be caused by injuries, trauma, infectious, and non*-*infectious factors, which can affect bones, muscle, skin, or the nervous system (Bradshaw et al., 2002; Kierończyk et al., 2017). Previous studies have demonstrated that lameness can be painful; lame birds self*-*selected feeds with analgesics (Danbury et al., 2000) and analgesic treatment improved lame birds’ walking ability (McGeown et al., 1999; Caplen et al., 2013). In addition, severely lame birds have difficulty reaching the feeders and drinkers, leading to malnutrition and mortality (Bradshaw et al., 2002; Kierończyk, 2017). In the United States alone, economic losses caused by lameness and skeletal disorders have been estimated to cost producers more than $150 million USD per year (Kierończyk, 2017). Therefore, lameness represents both a welfare and economic issue in broiler production.

The most common disorders that affect broiler chickens’ walking ability include bacterial chondronecrosis with osteomyelitis, tibial dyschondroplasia (**TD**), valgus-varus deformity, and contact dermatitis (Bradshaw et al., 2002). These abnormalities mainly affect the bones and joints of broiler chickens (Edwards and Veltmann, 1983; Julian, 1984; Bradshaw et al., 2002; Shim et al., 2012a,b), causing problems with skeletal or structural development. Unlike other conditions causing lameness, contact dermatitis is associated with inflammation and lesions of the skin rather than disturbances in the bone structure (Bessei, 2006). In broiler chickens, contact dermatitis is commonly found on the feet or hocks, being referred to as footpad dermatitis (**FPD**) and hock burns (**HB**), respectively (Hartcher and Lum, 2020).

Severe contact dermatitis can be associated with pain and increased propensity to secondary bacterial infections, which may aggravate leg disorders (Bessei, 2006; Hartcher and Lum, 2020). Even though the low locomotor activity and prolonged time spent sitting are not a welfare problem per se, they can cause or aggravate the risk of contact dermatitis, especially if the birds are raised in poor environmental conditions, with wet litter and high ammonia levels (Robins and Phillips, 2011). In addition, increased locomotor activity is associated with improved bone development and quality, potentially decreasing the propensity for leg disorders (Reiter and Bessei, 1998; Bizeray et al., 2000; Hartcher and Lum, 2020).

The combination of selection for accelerated growth and low locomotor activity is considered a risk factor for the development of skeletal disorders and skin lesions that may impair birds’ walking ability (Bradshaw et al., 2002). Because of the large influence of lameness on both welfare and economics, breeding companies have incorporated skeletal health traits into breeding programs to mitigate the occurrence of bone abnormalities and lameness in FG strains (Angel, 2007; Whitehead, 2007; Kapell et al., 2012). However, recent studies have estimated moderate to severe gait impairment in about 14 to 30% of FG broiler chickens, with 3.3% of the birds being almost unable to walk based on gait scores (Knowles et al., 2008; Bassler et al., 2013; Kittelsen et al., 2017; Vasdal et al., 2018), suggesting that lameness is still an ongoing issue.

The Bristol 6-point gait scoring system developed by Kestin et al. (1992) is the most widely used methodology to investigate walking ability and lameness in broiler chickens (Caplen et al., 2012). Scores equal to or greater than 3 are assumed to be painful, indicating that the welfare of birds may be compromised (McGeown et al., 1999; Weeks et al., 2000; Knowles et al., 2008; Caplen et al., 2013; Hartcher and Lum, 2020). Despite the widespread use of the Bristol gait scoring scheme to assess lameness in commercial broiler flocks at farm and research settings, this method provides a subjective estimation of birds’ walking ability and requires observers to classify the different degrees of gait problems. This can be difficult when comparing strains with vastly different phenotypes, as differences in motivation to walk (Bizeray et al., 2000; Bokkers and Koene, 2004), body conformation (Corr et al., 2003a), and temperament (Bizeray et al., 2000; Castellini et al., 2002; Bokkers and Koene, 2004; Bessei, 2006; Dixon, 2020) may influence birds’ locomotion and/or gait. Therefore, other tests have been studied to assess lameness and leg health in broiler chickens.

Two validated behavioral tests for lameness and mobility include the latency-to-lie (**LTL**) test, developed by Weeks et al. (2002), and the group obstacle test, developed by Caplen et al., (2014). The LTL assesses the length of time the birds will stand to avoid lying in shallow water, which is considered to be a novel experience and an aversive stimulus. The group obstacle test measures the frequency with which birds will cross an obstacle placed in their home pen to obtain access to water and feed, critical resources that are located on opposite sides of the obstacle. Both tests are correlated with the traditional Bristol gait scoring system, with lame birds (high gait score) lying down earlier in water (Weeks et al., 2002; Caplen et al., 2014) and showing fewer obstacle crossings (Caplen et al., 2014) compared to sound birds (low gait score), in the LTL and group obstacle test, respectively. However, these tests were mainly conducted using FG chickens (Berg and Sanotra, 2003; Caplen et al., 2014) or they compared a limited number of SG strains (Singh et al., 2021). Therefore, there is scarce information on the possible behavioral differences between FG and SG birds using objective tests that assess walking ability and lameness in broiler chickens.

Due to the welfare implications associated with fast growth rate, especially those causing lameness and impaired locomotion, there is an increasing interest in the use of SG strains in commercial broiler production. Previous comparisons between strains diverging in growth rate demonstrated better walking ability and a lower incidence of contact dermatitis in slower growing (**SG**) strains compared to FG chickens (Castellini et al., 2016; Dixon, 2020). However, there is a scarcity of studies comparing strains differing in growth rates under the same confined conditions. In addition, considering the continuous and dynamic changes in genetic selection, previous studies comparing SG and FG strains may not accurately reflect the genetics of modern broiler chickens (Rayner et al., 2020). Although the term “slow growing” commonly refers to birds with reduced growth rate and feed efficiency compared to FG birds, it encompasses a heterogeneous group of birds that represents various rates of growth (Doğan et al., 2019; Mancinelli et al., 2020).

Therefore, the aim of this study was to investigate the differences in mobility and contact dermatitis between 2 FG and 12 SG strains of broiler chickens raised under the same conditions and processed at similar market weights. The LTL and group obstacle tests were used to investigate leg strength and mobility of both FG and SG birds. We predicted that SG birds would have better leg health, as indicated by longer time spent standing in water, more obstacle crossings, and lower incidence and severity of contact dermatitis compared to FG chickens.

## MATERIALS AND METHODS

### Hatching and Husbandry

The procedures carried in this study were approved by the University of Guelph's Animal Care Committee (AUP #3746) and were in accordance with the Canadian Council for Animal Care's guidelines (CCAC, 2009).

This study is part of a multidisciplinary project that investigated production performance, meat quality, behavior, physiology, bone traits, health, and inactivity levels of FG and SG strains selected for distinct growth rates, described in other associated papers. The complete details regarding the overall methodology of this multidisciplinary study (e.g., incubation conditions, animal handling, husbandry, management, and housing) are available elsewhere (Torrey et al., 2021).

In short, the study encompassed 8 trials conducted at the Arkell Poultry Research Station (Guelph, ON, Canada). Each trial represented a typical broiler production cycle, from incubation and hatch to slaughter, with 5 to 7 strains tested per trial, and a total of 14 strains (2 FG and 12 SG) tested throughout the study. In each trial, fertile eggs from each strain were incubated simultaneously under standardized conditions at the federally inspected facility at Arkell Poultry Research Station. In total, 7,216 birds were reared over 8 trials in a single room, containing 28 floor pens (160 cm × 238 cm; width × length) with an expected stocking density of 30 kg/m^2^ at both TWs obtained through reducing the number of birds (e.g., thinning) at specific time points to maintain as similar a stocking density among strains as possible. Details about the stocking density and overall methodology of the study are described by Torrey et al. (2021).

The room was divided into 4 blocks according to the location of the pens to account for micro climate differences detected in pilot studies. Each strain was tested in up to 3 trials, with 4 pens per trial representing each block of the room, totaling 12 pens per strain, except for strains G and M. Due to the low availability of fertile eggs, strain G was tested in 4 production cycles, with 2 pens represented in each of 2 trials totaling 4 pens, while the remaining 8 pens were equally divided into 2 trials (4 pens per trial). Strain M was tested in 2 trials (8 pens) due to the limited availability of fertile eggs.

In each pen, a total of 44 birds were placed. The birds were vent sexed at the hatch to maintain sex balance, with 22 males and 22 females per pen. The group weight of each pen was obtained to keep a similar initial BW across the pens of each strain. From each pen, 12 birds (6 males and 6 females), used as focal birds, were individually weighed, wing tagged, and marked with livestock paint for identification purposes. These focal birds were used to assess behavioral, health, meat quality, and physiological parameters described in other studies to be published. All the birds received vaccines against infectious bronchitis, coccidiosis, and Marek's disease (Torrey et al., 2021).

In total, 164 groups of birds were reared in 28 pens over a 2-yr period. Each pen contained 5 nipple drinkers and a hanging round feeder (diameter: 33.75 cm). The pens were enriched with a 30 cm high-raised platform attached to an angled 25° ramp, a hanging round scale (diameter: 50.8 cm), a quarter of a mineral PECKstone (Protekta, Lucknow, Ontario, Canada), and a hanging nylon rope tied to strips of polyester as an oral enrichment. Softwood shavings were used as litter bedding and were removed and replaced at the end and beginning each trial, respectively. The addition of litter was not practiced in the same trial to simulate conventional poultry houses in Ontario, in which litter is only replaced prior to the placement of each new flock. Birds had ad libitum access to a 3 phase (starter, grower, and finisher) all–vegetable and antibiotic–free diet formulated for slow-growth. The feed type (grower and finisher) was switched when strains reached a similar feed intake compared to FG birds. Light intensity was kept at 20 lux. On the first 3 d, the lighting schedule was maintained at 23 h of light (L) and 1 h on the dark (D) to allow birds to locate food and water. Thereafter, a 16L:8D photoperiod was used, with 1 continuous dark period. At placement, room temperature was maintained at 32°C and gradually decreased as the birds aged, reaching 21°C at 5 wk of age.

Both FG and SG were processed at 2 target weights (**TWs**) based on their breeder's expected time to reach 2.1 kg (**TW 1**) and 3.2 kg (**TW 2**). Due to the differences in growth rate between FG and SG strains, birds were processed at different ages, with half of the pens of each strain being processed at each TW. At TW 1 and TW 2, FG strains were 34 d and 48 d of age, respectively, whereas SG strains were 48 d and 62 d. These processing dates were intended to allow us to evaluate the response variables at a similar BW (approximately 2.1 and 3.2 kg) and similar age (48 d). However, due the variation in growth rate among strains tested in the present study, the BW of strains ranged from 1,671 to 2,442 g and 2,603 to 3,485 g at TW 1 and TW 2, respectively in the LTL test. For the group obstacle test the BW of strains ranged from 1,494 to 2,253 g and 2,322 to 3,154 g at TW 1 and TW 2, respectively.

### Latency-to-Lie Test

**Figure 1:**
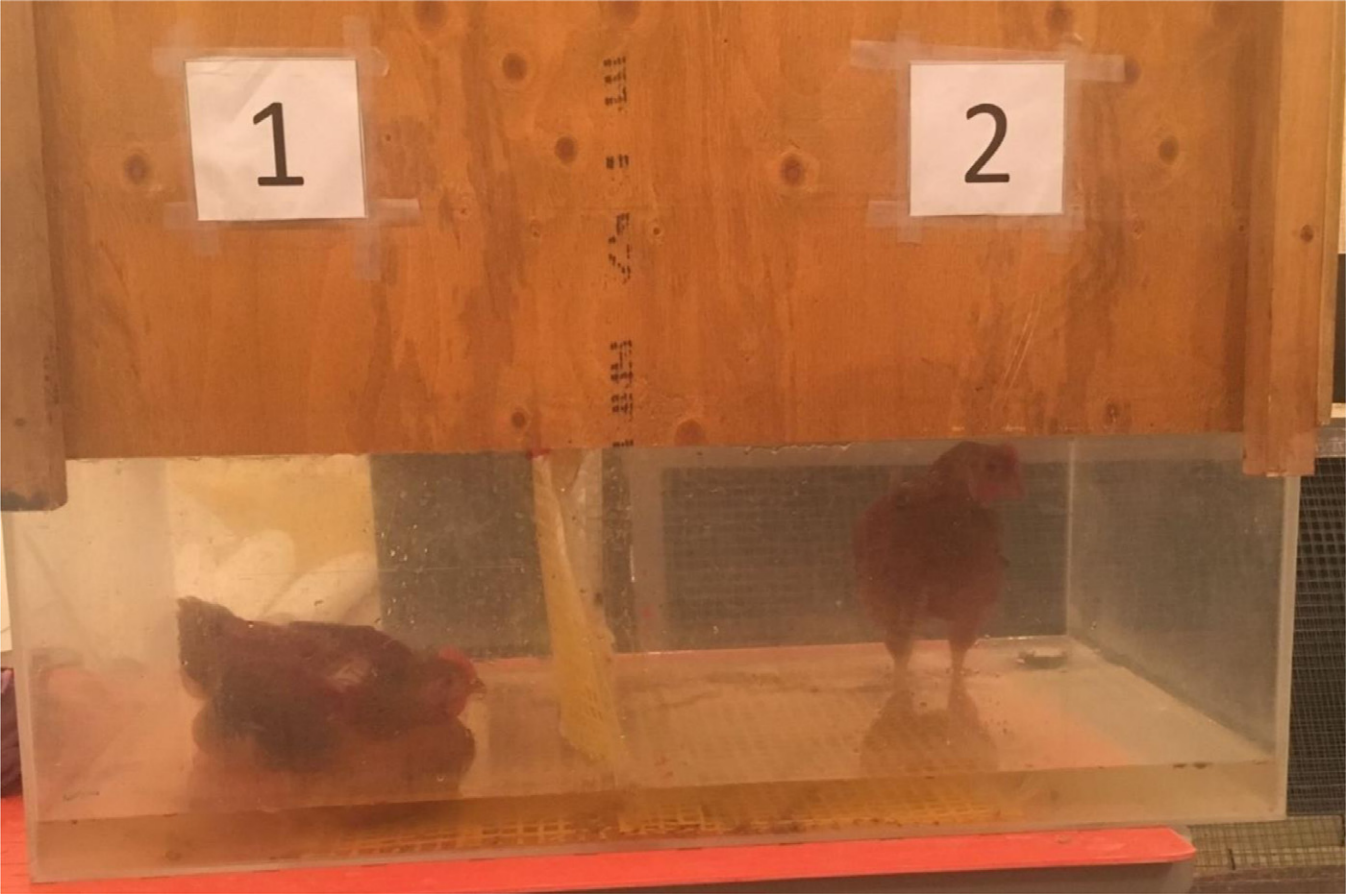
Broiler chickens during the latency-to-lie test. The tank was placed on top of heating mat to help maintain the water at 30°C to 32°C. The plexiglass portion of the tank was covered with a wood lid to prevent birds from flying out during the test. The birds were video recorded continuously during the test. The numbers on each side of the tank were used for identification purposes.

Four focal birds (2 males and 2 females) were tested 2 to 7 d prior to processing at each TW to assess leg strength. These birds were selected from the pens to be processed at each TW. Therefore, the test was conducted with different birds prior to TW 1 and TW 2, as half of the pens were processed at each TW. The LTL test followed a similar methodology described by Berg and Sanotra (2003) and (Caplen et al., 2014). The apparatus was a clear plexiglass tank (Figure 1; 98 × 48 × 103 cm; length × width × height) with a non-slip flooring and a plastic mesh partition that divided the test tank into 2 separate sections for simultaneously testing a pair of birds (Figure 1). A wood cover prevented the birds from flying out of the tank. Prior to testing, the container was filled with warm water (30°C to 32°C) at a depth of 4 cm. Water temperature was measured prior to testing additional birds to maintain a similar temperature for all the birds tested. Water was replaced as needed (i.e., if it became soiled due to birds defecating or it was not warm enough) prior to the placement of additional birds into the tank. For each test, 2 focal birds (1 male and 1 female) from the same pen were removed from their home pen, weighed, and simultaneously placed in the tank by 2 researchers, with the test starting when both birds were placed standing in the water. Because the birds were next to each other, removing 1 bird could affect the behavior of the adjacent bird. Therefore, both birds were kept in the tank and continuously recorded using a digital video camera (Sony Digital High-Definition Video Camera; HDR*-*CX405 and DCR*-*SR68 models; Sony, Japan) throughout the 10 min test. The time spent standing before lying down for the first time and the frequency of lying events per bird were recorded. Lying down was defined as a bird with its breast touching the water for at least 5 s, in which the last second of this 5 second (s) period was considered the latency-to-lie. In addition, a lying event was also counted if a bird laid down in water 3 consecutive times within a 20 s period, with each dip lasting less than 5 s and the third dip being considered the latency-to-lie. These short 3 consecutive dips within 20 s were considered as 1 lying event per bird while each time the bird laid down in water for at least 5 continuous seconds was counted as 1 lying event per bird. Therefore, the total frequency of lying events per bird was determined as the sum of these 2 lying events, although only about 10% of birds tested performed consecutive dips in water (i.e., at least 3 dips of less than 5 s each within 20 s). If a bird did not lie down in water throughout the test, the latency-to-lie was considered to be 600 s, which corresponds to the maximum time and cut-off point of the test. The latency-to-lie and frequency of lying events were determined for each bird tested.

### Group Obstacle Test

**Figure 2:**
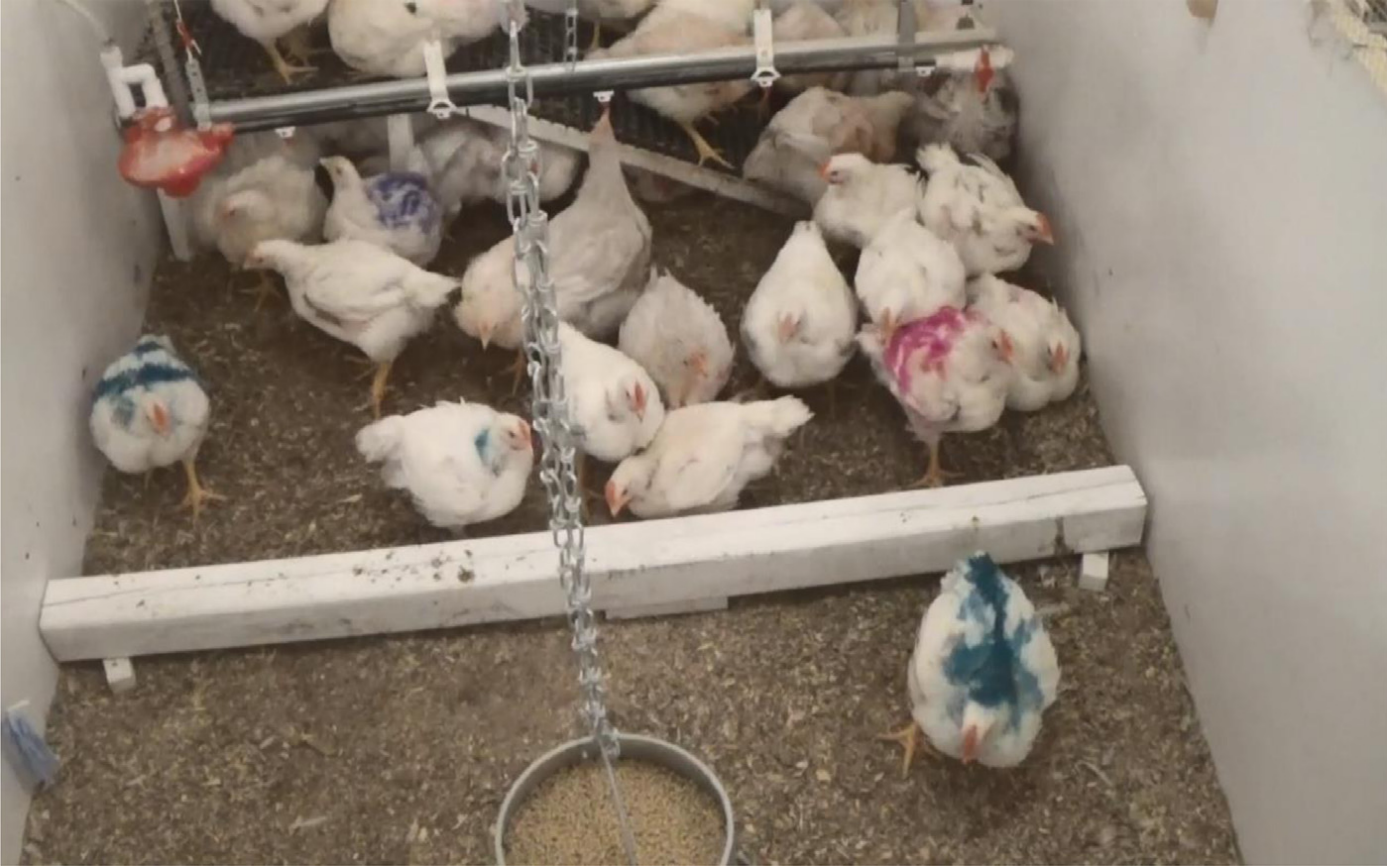
Broiler chickens during the obstacle test. The obstacle (white wooden barrier) was placed horizontally across the pen for 5 h, during which the birds were continuously recorded. Birds had to cross the obstacle to obtain access to the feeder (located in the front of the pen) or drinker (located in the back of the pen). Focal birds were identified by livestock paint on their backs.

The group obstacle test was conducted 3 to 7 d prior to processing and followed the methodology described by Caplen et al. (2014). For this test, a wooden barrier (160 × 9 × 10 cm; length × width × height; painted white) was placed alongside 1 wall in each pen 24 h before the test to habituate the birds to the presence of the new object, without preventing access to the feeder and drinker. Prior to the test, 6 birds per pen (3 males and 3 females) were individually marked and weighed. Four of these birds (2 males and 2 females) were the same birds used for the LTL test, whereas the other 2 birds were part of the focal birds selected at hatch in order to represent a larger proportion of the pen. Similar to the LTL test, the birds were selected from the pens to be processed at each TW, resulting in different birds being tested prior to TW 1 and TW 2. The LTL and group obstacle tests were conducted 4 to 7 d apart to allow the birds to recover, preventing a possible interaction between the tests. After the birds were weighed, the feeder was removed from each pen for a 1 h period to increase the birds’ motivation to obtain access to feed at the beginning of the test. Birds had free access to water during this period. After completion of the 1 h feed withdrawal (from the removal of feed of the last pen tested), 1 researcher used a board to corral the birds to the back of the pen, which contained the drinker line. The obstacle was placed horizontally across the pen, creating a barrier between the feeder and drinker (Figure 2). Therefore, the obstacle required the birds to step up and over it to reach the feed or water, which were located on opposite ends of the pen. The feeder was returned to the pen after placement of the obstacle. After the obstacle and feeder were placed into the pens, birds were continuously recorded for 5 h using the same equipment as in LTL test, positioned in front of each pen and angled towards the obstacle to allow visualization of both sides (feeder and drinker). The experimenter left the room after all the pens were set up (i.e., placement of feeders, obstacle, and video camera). The latency to first cross the obstacle and the total number of crossings (a combination of step*-*up and step*-*down towards the feeder or drinker side) per focal bird were tallied.

### Litter Moisture Content

On d 14, 28, 42, and 56, litter samples were collected from each pen to obtain an estimate of the litter moisture content. After each processing, litter was collected from the pens with birds remaining. Therefore, on d 14 and 28, litter was collected from all the pens, whereas on d 42 and 56, fewer pens were analyzed due to the different processing ages of FG and SG strains, as previously described. Because FG birds were processed at 34 d and 48 d, there are no data for this category at 56 d.

A square metal box (10 × 10 × 10 cm) was used to collect litter from 5 pre*-*determined locations in each pen, accounting for the left and right (front and back of the pen) and middle area, avoiding areas under the drinkers. The samples from the different locations were pooled and placed into sealed plastic bags identified with the pen number. Next, the samples were transferred to a dry bucket and thoroughly mixed until homogenized and a representative sample that ranged from 100 to 120 g was obtained and placed into pre-weighed aluminum containers. The total initial weight (litter + aluminum container) was recorded, the samples were dehydrated for 24 h at 65°C, then the final weight was recorded (Arrazola et al., 2019). Litter moisture content was estimated by dividing the water lost (difference between initial weight and final weight) by the initial weight.

### Footpad Dermatitis and Hock Burns

One day prior to processing (33, 47, or 61 d), 22 birds per pen (11 males and 11 females) were assessed to determine the prevalence and severity of FPD and HB using a 5 point scale from 0 to 4, as described by the Welfare Quality (2009) Protocol with 0 representing no lesions and 4 representing severe lesions. These birds included the 12 focal birds and 10 randomly selected birds (5 males and 5 females) to represent a large percentage (50%) of the pen. Because a low incidence of HB was observed in pilot studies and the first 3 trials (personal observation; data not analyzed), the assessment of HB was only conducted in trials 4 to 8. Therefore, strains D, H, and M were not represented in the HB evaluation, because these strains were only tested in the first 3 trials.

Both right and left feet and hocks were evaluated, and the highest score of the 2 was recorded. For data analyses, scores were categorized as 0*-* no lesions (score 0), 1*-*mild lesions (scores 1 and 2 combined) and 2 severe lesions (scores 3 and 4 combined). This classification was adapted from the National Chicken Council (2017), in which lesions covering less than 50% of the footpad (scores 1 and 2 in our scoring system) are classified as “pass” or acceptable, while lesions covering more than 50% of the footpad (scores 3 and 4 in our scoring system) are classified as “fail” or unacceptable. A similar classification was used for HB lesions, where scores 0 to 5 were categorized as to 0 no lesions, 1 mild lesions (red /light brown and superficial lesions) and 2 severe lesions (black and/or deep ulcers). The total incidence of birds exhibiting any FPD and HB and the incidence of birds exhibiting severe lesions (scores 3 and 4 combined) of FPD and HB were determined on a pen basis and reported as a percentage of total birds evaluated.

### Statistical Analyses

To facilitate analysis, strains were categorized into 4 groups based on their realized growth rate to TW 2 (48 d for FG and 62 d for SG strains, respectively, Torrey et al., 2021). The 14 strains were categorized as conventional (CONV; strains B and C; ADG_0_*_-_*_48_ = 66.0–68.7 g/d), fastest slow-growing (FAST; strains F, G, I, and M; ADG_0_*_-_*_62_ = 53.5–55.5 g/d), moderate slow-growing (MOD; strains E, H, O, and S; ADG_0_*_-_*_62_ = 50.2–51.2 g/d) and slowest slow-growing (SLOW; strains D, J, K, and N; ADG_0_*_-_*_62_ = 43.6–47.7 g/d). Comparisons among and within categories were conducted for all variables analyzed to assess differences among strains differing in growth rates and to compare strains with similar growth rates, respectively. Data were analyzed as an incomplete block design, using generalized linear mixed models (**GLIMMIX**) in SAS, version 9.4 (SAS Institute Inc 2018), with pen considered as the experimental unit. The random effects for all models included trial (i.e., each of the 8 production cycles) and block nested within trial. For all of the variables analyzed, except litter moisture, 2 models were used to assess the differences at a similar BW (*TW model*) and similar age (*Age model*). The main effects of the TW model included category, strain nested within category, TW, and sex. The interactions between category × TW, category × sex, category × sex × TW, strain (category) × TW, strain (category) × sex, and strain (category) × sex × TW were tested and included in the model if significant. This model allowed the determination of the effect of BW on leg strength (LTL test), number of obstacle crossings (group obstacle test), and incidence of contact dermatitis. The age model allowed the evaluation of all the strains at approximately 48 d, which corresponded to TW 1 and TW 2 for SG and FG strains, respectively. In the age model category, strain (category), and sex were included as main effects. The interactions between category × sex or strain (category) × sex were tested and kept in the model if significant.

Because litter moisture was assessed at different ages, the day of collection (14, 28 and 42 d) was used as a repeated measure in the *Litter moisture by age model*. The interactions between category × age and strain (category) × age were included as main effects and kept in the model if significant. This model included an ARH (1) structure, selected based on fit statistics with the lowest Akaike information criterion value. Because CONV birds were processed at d 34 and 48 at TW 1 and TW 2, respectively, the litter moisture was determined at d 56 only in the remaining SG birds. Therefore, an additional model (*Litter moisture at d 56*), including category and strain (category) was used to investigate the litter moisture content at d 56 for SG strains.

Contrast statements were used to identify differences between categories and between strains within categories. For multiple comparisons, *P-*values were adjusted using Tukey adjustment. Residuals were checked for normality using quantile*-*quantile plots and the Shapiro*-*Wilk test. Linearity, randomness, and homogeneity of residuals were assessed using scatterplots and boxplots of studentized residuals. Residual analyses were used to select the most appropriate model that met all the model assumptions. The Gaussian distribution was used by default if all of the model assumptions were met. For the total incidence of FPD (TW model), the number of lying events in the LTL test (TW model), and latency to cross the obstacle (TW and age models), binary, Poisson, and lognormal distributions were used, respectively, to meet the model assumptions. The main effects of sex and TW are not included in data tables and figures but are described with respective *P-*values in the results section if significant.

Due to the potential impacts of contact dermatitis on the variables evaluated in the LTL and group obstacle tests (Caplen et al., 2014), Spearman's rank correlation coefficients were used to investigate the relationships between the severity of FPD and HB with the LTL and group obstacle tests for each category. In addition, the correlation between the LTL and group obstacle tests were also investigated to determine possible relationships between the tests for each category. Correlation coefficients were classified as weak (r_s_ < |0.35|), moderate (r_s_ |0.35| ≤ r_s_ < |0.67|), or strong (r_s_ ≥ |0.68|) (Bohrer et al., 2018). For all tests performed, statistical significance was considered at *P* < 0.05.

**Figure 3:**
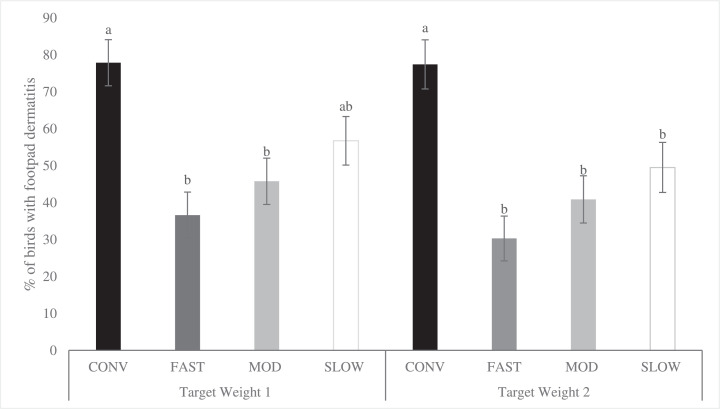
Effects of category on the total incidence of footpad dermatitis (LS-means ± SEM) at Target Weight 1^1^ and Target Weight 2^2^. At Target Weight 1, CONV and other categories were 34 and 48 d of age, respectively. At Target Weight 2, CONV and other categories were 48 and 62 d, respectively. Within Target Weight, columns with different superscripts differ (*P* < 0.05). ^1^ Number of birds per category evaluated at Target Weight 1: CONV: n = 273, FAST: n = 487, MOD: n = 547, SLOW: n = 528. ^2^ Number of birds per category evaluated at Target Weight 2: CONV: n = 220, FAST: n = 460, MOD: n = 500, SLOW: n = 504.

**Figure 4:**
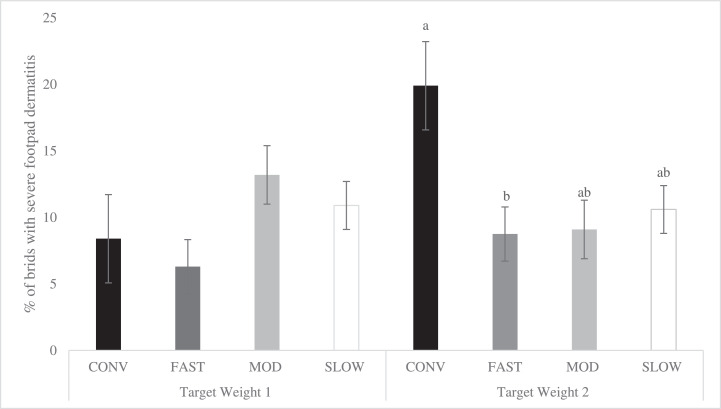
Effects of category on the total incidence of severe scores of footpad dermatitis (LS-means ± SEM) at Target Weight 1^1^ and Target Weight 2^2^. At Target Weight 1, CONV and other categories were 34 and 48 d of age, respectively. At Target Weight 2, CONV and other categories were 48 and 62 d, respectively. Within Target Weight, columns with different superscripts differ (*P* < 0.05). ^1^ Number of birds per category evaluated at Target Weight 1: CONV: n = 273, FAST: n = 487, MOD: n = 547, SLOW: n = 528. ^2^ Number of birds per category evaluated at Target Weight 2: CONV: n = 220, FAST: n = 460, MOD: n = 500, SLOW: n = 504.

**Figure 5:**
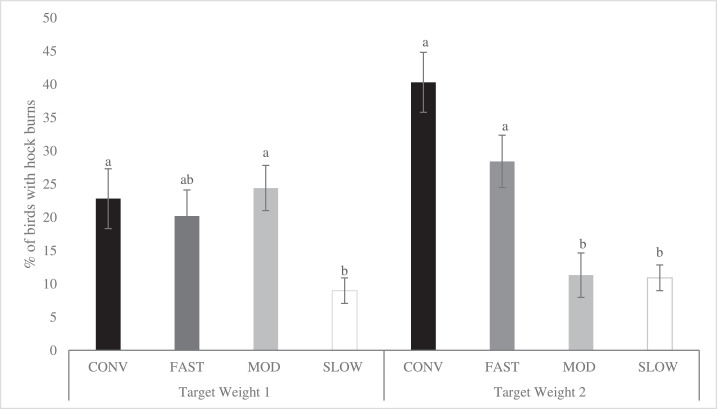
Effects of category on the total incidence of hock burns (LS-means ± SEM) at Target Weight 1^1^ and Target Weight 2^2^. At Target Weight 1, CONV and other categories were 34 and 48 d of age, respectively. At Target Weight 2, CONV and other categories were 48 and 62 d, respectively. Within Target Weight, columns with different superscripts differ (*P* < 0.05). ^1^ Number of birds per category evaluated at Target Weight 1: CONV: n = 112, FAST: n = 354, MOD: n = 352, SLOW: n = 396. ^2^ Number of birds per category evaluated at Target Weight 2: CONV: n = 131, FAST: n = 352, MOD: n = 353, SLOW: n = 395.

Due to the large number of strains tested and lack of significant differences among strains within category for most of the variables evaluated, differences among strains at each TW are included in the Supplementary Material (Supplementary Tables 1–4 and Figure 1, Figure 2, Figure 3, Figure 4, Figure 5) and are described in the results section only if trends or significant differences were found. This lack of significant differences among strains despite the large numeric differences were probably due to the small sample size and large variation among strains, leading to low statistical power **(**1-β **<** 0.65). Significant interactions between category, sex, and TW are also provided in Supplementary Material (Supplementary Table 5). Differences between sex and TWs are described in the text if significant. Overall, the main effect of TW is included in the results section but not discussed separately from category due to the interaction between these factors on most of the variables evaluated.

## RESULTS

### Differences among Categories, Strains, and Sexes at TW 1 and TW 2

#### Body Weight at LTL Test

As expected, BW was heavier in birds evaluated for the LTL at TW 2 compared to TW 1 (*P* < 0.001; TW 1 = 2,015 ± 24.5 g, TW 2 = 3,030 ± 25.7 g), which was consistent in all categories. Body weights differed by category at both TWs, with CONV and SLOW birds being lighter than FAST and MOD birds (*P* < 0.001). The FAST and MOD birds had differences in BW among strains (Supplementary Tables 2 and 3). Among FAST birds (Supplementary Table 2), strain M was lighter (*P* < 0.010) than the other FAST strains at TW 1, while at TW 2 no difference in BW was observed. Among MOD birds (Supplementary Table 3), strain H was lighter than the remaining strains at TW 1 while at TW 2 strain H was lighter than strains E and O (*P* < 0.004). Overall, males were heavier than females (*P* < 0.001; 2,777 ± 21.5 g vs*.* 2,267 ± 22.7 g). However, sex interacted with category (*P* = 0.001, Supplementary Table 5). At TW 1, for both females and males, CONV and SLOW were lighter than MOD and FAST birds. However, at TW 2, CONV females were lighter than FAST females, yet similar to MOD females, whereas CONV and SLOW males were similar yet both categories were lighter than FAST and MOD males.

#### Latency-to-Lie

Time spent standing in water before lying down (i.e., LTL) was longer at TW 1 (*P* = 0.042; 450.8 ± 11.96 s) than TW 2 (413.2 ± 12.78 s). However, TW interacted with category, indicating that differences in LTL between categories depended on TW (Table 1; *P* = 0.016). At TW 1, CONV and SLOW birds remained standing longer than FAST birds. At TW 2, CONV and FAST birds had a shorter time standing in the water than SLOW birds, while MOD birds did not differ from the other categories at either TW. There was no difference in LTL among strains within category at either TW (all pairwise comparisons among strains within category *P* > 0.171 for both TWs). Overall, standing time in shallow water was shorter (*P* ˂ 0.001) in males (373.9 ± 11.15 s) than in females (489.9 ± 12.83 s). However, there was a category × TW × sex interaction (*P* = 0.001; Supplementary Table 5). At TW 1, LTL was affected by sex in FAST and MOD categories, whereas at TW 2, all the categories were affected by sex, with males standing for less time in water than females. At both TW 1 and TW 2, no difference in LTL was observed between females across the different categories. At TW 1, CONV and SLOW males had greater values than FAST and MOD males, whereas at TW 2, SLOW males had greater values than males from the remaining categories.

**Table 1:** Effect of category on body weight (BW), latency-to-lie (LTL), and group obstacle test (LS means ± SEM) in the week prior to Target Weights 1 and 2. At Target Weight 1, CONV and other categories were 34 and 48 d of age, respectively. At Target Weight 2, CONV and other categories were 48 and 62 d of age, respectively.

	Category				
Variable	CONV	FAST	MOD		SLOW
Target Weight 1					
BW (g)- LTL test^1^	1,731 ± 55.9^b^	2,281 ± 45.6^a^	2,140 ± 44.1^a^		1,908 ± 46.5^b^
LTL (s)^2^	499.9 ± 31.19^a^	390.9 ± 23.01^b^	410.8 ± 21.38^ab^		489.7 ± 22.34^a^
% of birds lying per pen	26.3 ± 7.38^c^	57.8 ± 5.44^a^	50.3 ± 5.20^ab^		35.1 ± 5.32^bc^
Lying events per bird	0.57 ± 0.132^b^	1.08 ± 0.170^a^	0.78 ± 0.122^ab^		0.54 ± 0.100^b^
BW (g)- obstacle test^3^	1,792 ± 50.3^b^	2,008 ± 41.7^a^	1,896 ± 38.1^ab^		1,585 ± 34.9^c^
No. of obstacle crossings^4^	8.03 ± 0.702^b^	7.76 ± 0.558^b^	8.67 ± 0.638^b^		11.13 ± 0.639^a^
Latency to cross (s)	736.9 ± 162.12	1,084 ± 198.6	1,246 ± 178.9		985.8 ± 199.22
**Target Weight 2**					
BW (g)- LTL test	2,828 ± 61.3^b^	3,332 ± 48.5^a^		3,179 ± 45.27^a^	2,781 ± 46.8^b^
LTL (s)^5^	350.1 ± 32.25^b^	378.2 ± 24.18^b^		422.4 ± 22.96^ab^	479.5 ± 22.88^a^
% of birds lying per pen	59.2 ± 8.04	55.9 ± 5.92		47.7 ± 5.51	35.8 ± 5.59
Lying events per bird	1.30 ± 0.265^a^	1.19 ± 0.193^a^		0.69 ± 0.119^ab^	0.49 ± 0.096^b^
BW (g)- obstacle test	2,640 ± 50.3^b^	3,019 ± 42.4^a^		2,796 ± 38.1^b^	2,423 ± 35.8^c^
No. of obstacle crossings^6^	5.22 ± 0.698^b^	6.40 ± 0.567^b^		7.18 ± 0.639^ab^	9.15 ± 0.644^a^
Latency to cross (s)	2,148 ± 427.9	1,597 ± 258.7		1,778 ± 328.9	888.6 ± 124.03

a-c Different superscripts within the same row represent differences among categories (*P* < 0.05).

1 BW from focal birds tested in the latency-to-lie test. Birds were weighed on the same day the test was conducted.

2 Latency- to-lie per focal bird. Number of birds per category tested in the latency-to-lie test at Target Weight 1: CONV: n = 69, FAST: n = 106, MOD: n = 123, SLOW: n = 115.

3 BW from focal birds tested in the group obstacle test. Birds were weighed on the same day the test was conducted.

4 Number of obstacle crossings per focal bird. Number of birds per category tested in the group obstacle test at Target Weight 1: CON: n = 71, FAST: n = 130, MOD: n = 144, SLOW: n = 144.

5 Latency-to-lie per focal bird. Number of birds per category tested in the latency-to-lie test at Target Weight 2: CONV: n = 54, FAST: n = 95, MOD: n = 103, SLOW: n = 103.

6 Number of obstacle crossings per focal bird. Number of birds per category tested in the group obstacle test at Target Weight 2: CONV: n = 73, FAST: n = 126, MOD: n = 144, SLOW: n = 138.

#### Percentage of Birds that Laid Down in Water at the LTL Test

Category interacted with TW to influence the percentage of birds lying down in water (Table 1; *P* = 0.029). At TW 1, similar percentages of FAST and MOD birds laid down in water and more FAST birds laid down than CONV and SLOW birds. At TW 2, there was a tendency for more CONV (*P* = 0.067) and FAST (*P* = 0.083) birds to lie down than SLOW birds. Despite the large numeric differences, the percentages of birds lying down in the water did not significantly differ among strains within the same category (all pairwise contrasts between strains within category *P* > 0.581 at both TWs). The percentage of birds lying down in the water was greater (*P* < 0.001) in males (58.5 ± 2.71%) compared to females (33.53 ± 2.95%). However, an interaction between sex, category, and TW affected the percentage of birds lying in water (Supplementary Table 5). There was no difference in the percentage of birds lying in water among females, while among males there was an effect of category at both TWs. At TW 1, there were lower percentages of CONV and SLOW males lying down than FAST and MOD males, whereas at TW 2, a lower percentage of SLOW males laid down compared to males in the other categories.

#### Frequency of Lying Down Events Per Bird at the LTL Test

The interaction between category and TW affected the number of times the birds laid down in water (Table 1; *P* = 0.047). At TW 1, CONV and SLOW birds laid down in the water less often than FAST birds, while at TW 2, CONV and FAST birds laid down more times than SLOW. At both TWs, MOD birds did not differ from the remaining categories. At both TWs, there was no difference in the frequency of lying events among strains within category (all pairwise *P* > 0.615 for comparisons among strains within category at TW 1 and TW 2). Sex affected the number of times lying down in water, with males (*P* < 0.001; 1.07 ± 0.081 times per bird) lying down more often than females (0.56 ± 0.056 times per bird).

#### Body Weight at the Group Obstacle Test

As expected, birds tested in the obstacle test at TW 1 (*P* < 0.001; 1,820 ± 20.9 g) were lighter than those tested at TW 2 (2,719 ± 21.0 g). At TW 1, CONV and SLOW were lighter than FAST birds, while MOD birds were similar to CONV and FAST, yet heavier than SLOW birds (*P* < 0.001 Table 1). At TW 2, FAST birds had the heaviest BW, while CONV and MOD birds were similar and greater than SLOW birds. Within categories, MOD strains differed in BW only at TW 1 (see Supplementary Table 3), with strain E being heavier (*P* = 0.008) than strain S, while strains H and O did not differ from the remaining MOD strains. Heavier (*P* < 0.001) BW in males (2,469 ± 16.9 g) compared to females (2,070 ± 17.6 g) was consistent in all categories and strains.

#### Frequency of Obstacle Crossings Per Bird at the Group Obstacle Test

Overall, birds evaluated at TW 1 crossed the obstacle more often (*P* < 0.001; 8.9 ± 0.32 per bird) compared to those tested at TW 2 (6.9 ± 0.32 per bird). Nevertheless, differences among categories depended on TW (Table 1; *P* < 0.001). At TW 1, SLOW birds made the greatest number of crossings, while no difference was observed among the other categories. At TW 2, CONV and FAST birds made fewer crossings than SLOW, whereas MOD birds did not differ from the other categories. There was no effect of strain within categories on the total number of obstacle crossings (all pairwise comparisons among strains within category *P* > 0.602 at both TWs). Sex affected the total number of obstacle crosses (*P* = 0.028), with males (8.4 ± 0.27 per bird) crossing more than females (7.4 ± 0.29 per bird), which was consistent among all the categories.

#### Latency to Cross Obstacle for the First Time at the Group Obstacle Test

At TW 1, birds tended (*P* = 0.093) to have shorter latency to cross the obstacle (1052.7 ± 97.58 s) than at TW 2 (1531.3 ± 141.43 s). Although CONV birds tended to have greater latency to cross the obstacle than SLOW birds (*P* = 0.079) at TW 2, category (Table 1) and strain within category (*P* > 0.881) did not influence the latency to cross the obstacle (Supplementary Tables 1–4). Males had shorter latency to cross the obstacle than females (*P* = 0.018; 1184.5 ± 112.35 s vs*.* 1419.5 ± 132.99 s), with no interaction between sex and category (*P* = 0.167) or strain (*P* = 0.270).

#### Contact Dermatitis

The total incidence of FPD was affected by category (Figure 3; *P* < 0.001) at both TWs. At TW 1, CONV birds had a greater incidence of FPD than FAST and MOD birds, while SLOW birds did not differ from the other categories. At TW 2, CONV birds had the greatest incidence of FPD, with no difference observed among the SG categories. Despite the large numeric differences across strains within categories, the total incidence of FPD did not differ (*P* > 0.05 for all pairwise comparisons among strains within category at both TWs, see Supplementary Figure 1). Sex affected the incidence of FPD (*P* < 0.001), with fewer males (45.4 ± 3.04%) exhibiting FPD than females (59.6 ± 2.95%).

At TW 1, there was no difference in the incidence of severe FPD among categories. However, at TW 2, CONV had a greater incidence of severe FPD than FAST birds and tended to have greater incidence than MOD (*P* = 0.060) and SLOW birds (*P* = 0.094) (Figure 4). Within categories, there were no differences among strains at both TWs (*P* > 0.227 for all pairwise comparisons between strains within category at TW 1 and TW 2, Supplementary Figure 2). Sex (*P* = 0. 367) and TW (*P* = 0.243) did not affect the incidence of severe FPD.

Total incidence of HB was affected by category (*P* < 0.001). However, category interacted with TW (*P* = 0.011) as shown in Figure 5. At TW 1, CONV and MOD categories exhibited a similar percentage of birds affected by HB, which was greater than SLOW, while FAST did not significantly differ from the other categories. At TW 2, CONV and FAST birds had greater incidence of HB than MOD and SLOW birds, which had similar values. Within categories, CONV strains had a different incidence of HB at TW 1, with strain B being greater than strain C (*P* = 0.005, Supplementary Figure 3). At TW 2, no difference among strains within category were observed (*P* > 0.789 for all pairwise comparisons between strains within category at TW 2). The incidence of HB was affected by sex (*P* < 0.001), with more males (25.5 ± 2.87%) having HB than females (13.0 ± 0.017%).

**Figure 6:**
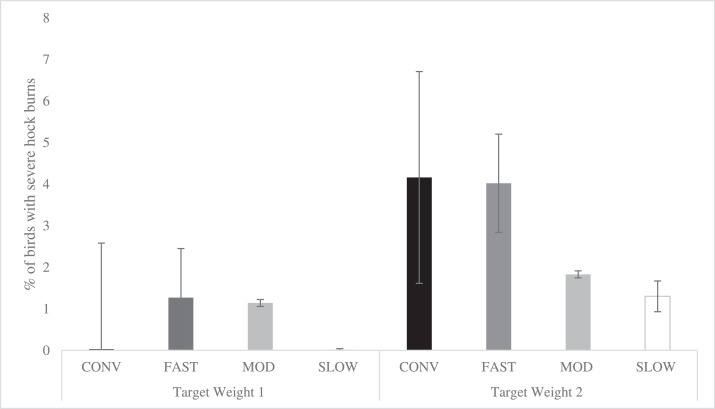
Effects of category on the total incidence of severe scores of hock burns (LS-means ± SEM) at Target Weight 1^1^ and Target Weight 2^2^. At Target Weight 1, CONV and other categories were 34 and 48 d of age, respectively. At Target Weight 2, CONV and other categories were 48 and 62 d respectively. ^1^ Number of birds per category evaluated at Target Weight 1: CONV: n = 112, FAST: n = 354, MOD: n = 352, SLOW: n = 396. ^2^ Number of birds per category evaluated at Target Weight 2: CONV: n = 131, FAST: n = 352, MOD: n = 353, SLOW: n = 395.

Birds processed at TW 1 had lower incidence of severe HB than those processed at TW 2 (*P* = 0.031; 0.61 ± 0.739% vs*.* 2.82 ± 0.739%). There was no effect of category (*P* > 0.129 for all pairwise comparison among categories at TW 1 and TW 2 Figure 6) or strain within category (*P* > 0.970 for all pairwise comparison among strains within category at TW 1 and TW 2, Supplementary Figure 4) in the incidence of severe HB lesions at both TWs. However, sex influenced the incidence of severe HB (*P* = 0.001), with more males having severe HB than females (2.57 ± 0.571% vs*.* 0.86 ± 0.570%).

### Effect of Category at a Similar Age

#### Latency-to-Lie and Group Obstacle Tests

Comparisons among categories at similar ages, which corresponds to 41 d and 46 d of age for the group obstacle and latency-to-lie tests, respectively, are described in Table 2. Category affected all the variables evaluated. The BW of birds differed among categories in both the LTL and group obstacle tests (*P* < 0.001), with CONV birds being heavier than the other categories, while FAST and MOD birds were similar and heavier than SLOW birds.

**Table 2:** Effect of category on body weight (BW) latency-to-lie (LTL), and group obstacle test (LS-means ± SEM) obtained at a similar age. Birds were tested at approximately 41 d and 46 d of age for the group obstacle test and latency-to-lie, respectively. Data corresponds to the week prior to Target Weight 1 and Target Weight 2 for slower-growing and CONV categories, respectively.

	Category			
Variable	CONV	FAST	MOD	SLOW
BW (g)- LTL test^1^	2,859 ± 66.0^a^	2,292 ± 44.3^b^	2,148 ± 43.3^b^	1,908 ± 44.5^c^
LTL (s)^2^	350.7 ± 34.84^b^	390.7 ± 24.47^b^	412.02 ± 23.20^ab^	490.1 ± 24.35^a^
% of birds lying per pen	60.0 ± 8.39^a^	58.9 ± 5.66^a^	51.0 ± 5.39^ab^	34.8 ± 5.50^b^
Lying events per bird	1.63 ± 0.281^a^	1.22 ± 0.197^ab^	0.89 ± 0.187^ab^	0.61 ± 0.196^b^
BW (g)- obstacle test^3^	2,670 ± 56.3^a^	2,003 ± 40.7^b^	1,905 ± 37.2^b^	1,590 ± 36.1^c^
No. of obstacle crossings^4^	5.21 ± 0.731^c^	7.79 ± 0.608^b^	8.69 ± 0.644^b^	11.11 ± 0.635^a^
Latency to cross (s)	2,148 ± 427.9^a^	1,084 ± 198.6^b^	1,246 ± 178.4^ab^	985.8 ± 195.73^b^

abc Different superscripts within the same row represent differences among categories (*P* < 0.05).

1 BW from focal birds tested in the latency to lie test. Birds were weighed on the same day the test was conducted.

2 Latency-to-lie per focal bird. Number of birds per category tested at a similar age: CONV: n = 54, FAST: n = 106, MOD: n = 123, SLOW: n = 115.

3 BW from focal birds tested in the group obstacle test. Birds were weighed on the same day the test was conducted.

4 Number of obstacle crossings per focal bird. Number of birds per category tested at a similar age: CONV: n = 73, FAST: n = 130, MOD: n = 144, SLOW: n = 144.

Category affected LTL (*P* < 0.003), with CONV and FAST birds having a shorter latency than SLOW birds. Similarly, a higher percentage of CONV and FAST birds laid down in water than SLOW birds (*P* = 0.001). The frequency of lying events was greater (*P* = 0.016) in CONV than SLOW birds, while FAST and MOD birds did not differ from CONV and SLOW birds.

The CONV birds had the lowest total frequency of obstacle crossings, followed by FAST and MOD, which did not differ from each other and were lower than SLOW birds. The latency to first cross the obstacle was greater in CONV birds compared to FAST and SLOW birds (*P* < 0.045), while MOD birds did not significantly differ from the other categories.

Differences in the LTL test between males and females at a similar age were similar to the differences previously described in the comparisons among categories at TW 1 and TW 2. Males were heavier (*P* < 0.001; 2,534 ± 27.13 g vs*.* 2,119 ± 28.2 g), had shorter LTL (*P* = 0.001; 375.2 ± 16.49 s vs*.* 447.0 ± 18.40 s), had a higher percentage of birds lying down in the water (*P* = 0.005; 58.7 ± 3.98% vs*.* 43.7 ± 4.27% s), and laid down in the water more times (*P* = 0.027; 1.29 ± 0.133 times vs*.* 0.89 ± 0.148 times) than females. However, when compared at a similar age, sex did not affect the frequency of obstacle crossings (*P* = 0.167; males = 8.5 ± 0.39 times vs*.* females = 7.9 ± 0.42 times). Males tended to take less time to first cross the obstacle than females at a similar age (*P* = 0.071; 1133.3 ± 141.81 s vs*.* 1419.8 ± 185.33 s). There was no interaction between sex and category or sex and strain for any variable evaluated at a similar age (*P* > 0.05).

#### Contact Dermatitis

**Figure 7:**
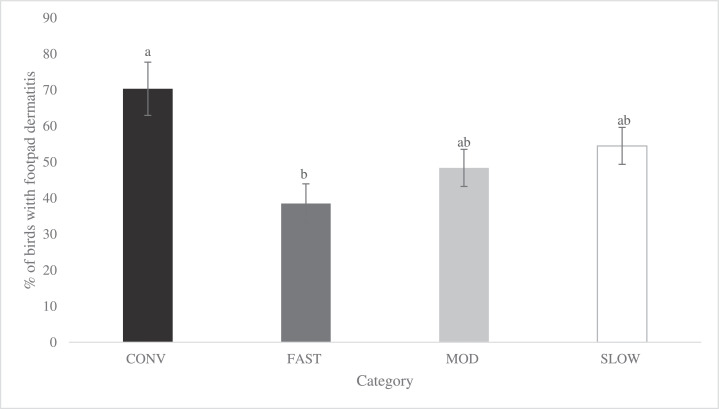
Effects of category on total incidence of footpad dermatitis (LS-means ± SEM) of broiler chickens at 48 d of age^1^. ^1^ Number of birds per category evaluated at a similar age: CONV: n = 220, FAST: n = 487, MOD: n = 547, SLOW: n = 528.

**Figure 8:**
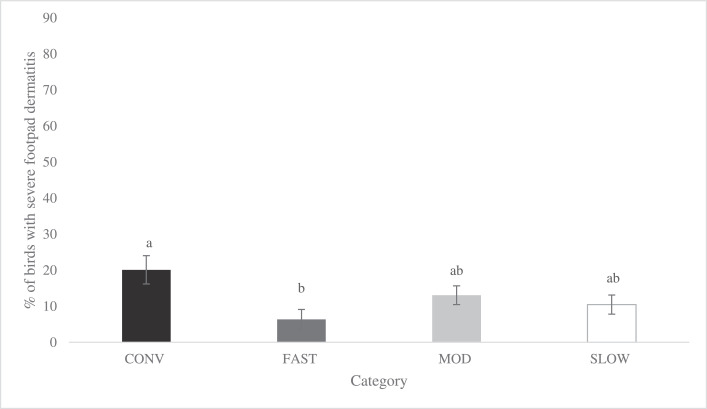
Effects of category on total incidence of severe footpad dermatitis (LS-means ± SEM) of broiler chickens at 48 d of age^1^. ^1^ Number of birds per category evaluated at a similar age: CONV: n = 220, FAST: n = 487, MOD: n = 547, SLOW: n = 528.

**Figure 9:**
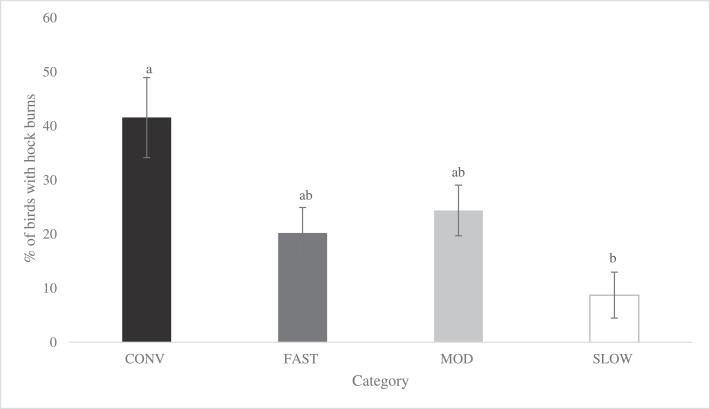
Effects of category on total incidence of hock burns (LS-means ± SEM) of broiler chickens at 48 d of age^1^. ^1^ Number of birds per category evaluated at a similar age: CONV: n = 131, FAST: n = 354, MOD: n = 352, SLOW: n = 396.

Category affected both FPD and HB when compared at a similar age. The CONV birds had a greater total incidence (Figure 7; *P* = 0.004) and severity (Figure 8; *P* = 0.026) of FPD compared to FAST birds, while MOD and SLOW birds were similar and did not differ from CONV and FAST birds. For HB, CONV birds had a greater total incidence (Figure 9; *P* = 0.001) compared to SLOW, whereas FAST and MOD birds did not differ from CONV and SLOW birds. Due to the low incidence of severe HB lesions in all categories (CONV: 4.16 %, FAST: 1.27%, MOD: 1.14%, SLOW: 0.01%), statistical analyses were not performed.

When compared at the same age, differences in contact dermatitis between the sexes followed a similar trend to the differences at the 2 TWs, with males exhibiting a lower total incidence of FPD than females (*P* < 0.001; 47.4 ± 3.11% vs*.* 58.3 ± 3.21%), whereas sex did not affect the incidence of severe FPD (P = 0.662; males: 12.9 ± 1.69%, females: 12.1 ± 1.79%). For HB lesions, males had a greater (*P* < 0.001) total incidence than females (32.1 ± 3.05% vs*.* 15.3 ± 3.08%).

#### Litter Moisture Content

**Figure 10:**
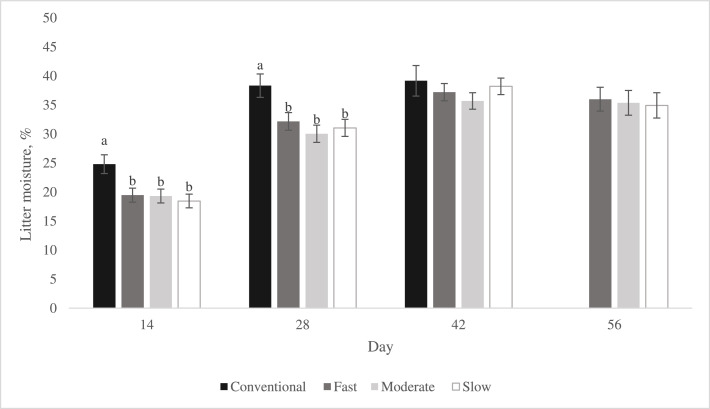
Effect of category on litter moisture (LS-means ± SEM) on day 14^1^, 28^2^, 42^3^ and 56^4^. CONV birds are not represented on d 56 because birds were processed at 34 and 48 d. Within age, columns with different superscripts differ (*P* < 0.05). ^1^ Number of pens per category at d 14: CONV: n = 24, FAST: n = 44, MOD: n = 48, SLOW: n = 48. ^2^ Number of pens per category at d 28: CONV: n = 24, FAST: n = 44, MOD: n = 48, SLOW: n = 48. ^3^ Number of pens per category at d 42: CONV: n = 12, FAST: n = 44, MOD: n = 48, SLOW: n = 48. ^4^ Number of pens per category at d 56: FAST: n = 22, MOD: n = 24, SLOW: n = 24.

As shown in Figure 10, litter moisture content differed among categories at d 14 and 28, with CONV having higher moisture than the SG categories (*P* < 0.003). However, at d 42, there was no difference among categories in litter moisture content. No difference among SG categories was observed at d 56 (Figure 10). Litter moisture differed within category for FAST and SLOW birds at d 42, but not at other ages. Among FAST birds, strain M had lower litter moisture than strains G and I (*P* < 0.022, Supplementary Figure 5 B) while strain F did not differ from the other FAST strains. Among SLOW strains, strain J had greater litter moisture content than strain D and K (*P* < 0.001, Supplementary Figure 5 D), whereas strain N was similar to the remaining SLOW strains. Litter moisture did not differ among strains within category for CONV and MOD birds at any age evaluated.

### Correlation among Contact Dermatitis, Latency-to-Lie, and Group Obstacle Test

For all the categories, there was no correlation between FPD scores and time spent standing in the LTL test or FPD scores and the number of times lying down in the water (*P* > 0.1226 for all categories, data not shown). For the obstacle test, there was a negative correlation between FPD scores and latency to first cross the obstacle for CONV birds only (*P* = 0.025; r_s_ = −0.264) suggesting a decrease in latency to cross as the FPD scores increased. In addition, a positive correlation was found between FPD scores and BW for CONV birds (*P* = 0.048; r_s_ = 0.233). The correlations between HB scores and the mobility tests were not consistent among the categories. While for CONV and SLOW birds there were no correlations between HB scores and any of the variables measured (data not shown), that was not the case for FAST and MOD birds. For FAST birds, there was a negative correlation between HB scores and time standing in water (*P* = 0.010; r_s_ = −0.264) and a positive correlation between HB scores and the number of times the birds laid down (*P* = 0.004; r_s_ = 0.292), which was also observed for MOD birds (*P* = 0.004; r_s_ = 0.290).

A strong negative correlation was found between time standing in the water and the number of times the birds laid down in the water for all the categories in the LLT test (*P* < 0.001; r_s_ < −0.85 for all categories, data not shown), whereas in the group obstacle test there was a moderate negative correlation between latency to the first cross and total obstacle crossings (*P* < 0.001; −0.3863 > r_s_ > −0.6138 for all categories, data not shown). No correlation between the variables measured in the LTL and group obstacle tests were observed for all the categories.

## DISCUSSION

The primary goal of this study was to investigate the differences in mobility, leg strength, and contact dermatitis among 14 strains of broiler chickens differing in growth rate when raised under similar rearing conditions and when those traits were compared at similar BWs and at a similar age. Many of the variables evaluated in this study were affected by growth rate category and/or BW at a similar TW and age, indicating their effects on traits associated with locomotion. However, the differences in FPD and HB lesions may be more associated with environmental conditions than growth rate, suggesting the importance of maintaining good litter quality to prevent the occurrence of contact dermatitis in both FG and SG birds.

### Latency-to-Lie Test

#### Effect of Category at Each TW

At TW 1, CONV and SLOW birds remained standing in the water for longer and had the lowest percentage of birds lying down and frequency of times lying down in water. These categories were also the lightest birds at TW 1, suggesting that differences in BW contributed more to performance in the LTL than did genetic potential for growth rate. The lower BW of ONV birds despite their greater growth performance was mainly due to the processing age that occurred earlier than the expected time for CONV strains to reach 2.1 kg (Santos et al., 2021a). On the other hand, the lower BW of SLOW birds is attributed to their reduced growth rate (ADG <50 g/d) compared to the other categories (Torrey et al., 2021). The longer LTL at TW 1 in CONV and SLOW birds indicates a better ability to support their BW (i.e., leg strength) compared to FAST birds. These results differ from those recently reported by Dixon (2020), who found a greater proportion of lower gait scores in SG birds, suggesting better walking ability in this group compared to 3 FG strains Kestin et al (2001). reported better gait scores in SG strains compared to FG strains when the birds were fed a similar diet. However, the same authors found that the effect of genotype disappeared when differences in BW were considered; this suggests that the variation in gait score in that study was mainly attributed to the differences in BW, similar to results reported here.

Although BW was likely the determining factor for the differences observed at TW 1, this was not the case at TW 2. At the heavier TW, CONV and SLOW birds were lighter than MOD and FAST birds, yet CONV and FAST birds spent less time standing and had a greater number of times lying down compared to SLOW birds. While the differences in time standing on water between FAST and SLOW birds may be attributed to the heavier BW of the former, SLOW and CONV birds had a similar BW at TW 2. Therefore, the differences in the LTL test between CONV and SLOW birds at TW 2 appear related to genetic potential for growth rates. This suggests that as the birds grow, the differences in leg strength between SG and FG genotypes may become more evident even when the birds are evaluated at a similar BW, indicating a negative effect of accelerated growth rate and potentially an effect of body conformation on leg strength. These results agree with other studies, in which SG birds showed better walking ability (as measured by gait score) than FG birds even when the evaluations were performed at a similar BW (Corr et al., 2003b Dixon, 2020; Rayner et al., 2020) or when differences in BW were taken into account (Kestin et al., 1999). A recent study by Singh et al. (2021) found a shorter standing time during the LTL test in FG compared to SG birds when both strains were evaluated at a similar BW of 2.0 to 2.2 kg, which overlaps with the expected BW of TW 1 (i.e., 2.1 kg) in the present study. The researchers suggested that this difference found in the LTL test could be attributed to the larger breast muscle of FG birds that may increase sternal mass and load, which consequently increases the metabolic costs associated with prolonged standing (Tickle et al., 2018), leading to a decrease in time standing for FG birds.

Although the LTL is associated with gait score (Weeks et al., 2002 Caplen et al., 2014) and validated by use of analgesics (Hothersall et al., 2016), the test relies on the notion that birds find sitting in water aversive. Aversion to water has also been used in behavioral tests with broiler breeders (Dixon et al., 2014). However, chickens’ aversion to sitting in water per se has not been directly validated. In a modified LTL test, in which no water was used and birds’ spontaneous LLT was measured in their home pens, Bailie et al. (2013) and Norring et al. (2019) reported shorter LTL (< 25 s) than those observed by Weeks et al. (2002), Berg and Sanotra (2003), and Caplen et al. (2014), in which birds’ LTL were as long as 600 to 900 s when water was used to increase birds’ motivation to stand. These differences in LTL obtained between the modified (no addition of water) and traditional LTL test (addition of water) suggest that water is likely considered an aversive stimulus to birds. Nevertheless, because these results were obtained from different studies, individual aversion to water was not assessed. Furthermore, these studies only tested FG birds. As such, differences in aversion to water in the LLT test among strains differing in growth rate is unknown and should be further investigated. Just as there may be genetic differences in fearfulness (Lindholm et al., 2017 Castellini et al., 2016), there could also be genetic variation in aversion to sitting in water. Therefore, similar to the gait score assessment, the LTL test may present its limitation when used to evaluate leg strength, as aversion to sit on water and motivation to stand may potentially differ among birds and strains.

Interestingly, from TW 1 to TW 2, CONV strains had a significant increase in percentage of birds lying down in water, shorter time standing, and tended to exhibit a greater number of times lying down in water. No significant changes or trends were observed for the SG categories as the birds grew from TW 1 to TW 2, despite the significant increase in BW in all categories. These results suggest that the detrimental effects of increasing age and BW were more evident in CONV birds, indicating potential negative effects of selection for growth on leg strength. However, because FG and SG birds were evaluated at different ages at TW 1 and TW 2, age-related changes in behavior and aversion to water should not be discarded. Furthermore, CONV birds had an increase in BW from TW 1 to TW 2 that was 14.85 to 17.70% greater than the remaining categories. Therefore, the sharp increase in BW observed in CONV birds from TW 1 to TW 2 may have exacerbated the differences obtained in the LTL as the birds grew.

#### Differences Among Categories at a Similar Age

Despite the differences in BW among categories, the time spent standing, percentage of birds lying down, and the number of lying events in the LTL test was similar in CONV, FAST, and MOD birds, while CONV birds differed from SLOW birds, indicating better leg strength in the latter. It is important to mention that SLOW birds were over 1-kg lighter than CONV birds at the same age. The differences between CONV and SLOW in the LTL test agree with many studies that suggest negative effects of increased BW on leg health (Kestin et al., 2001 Shim et al., 2012a, b; Dixon, 2020). However, our results suggest that at the same age, the effects of growth rate and increased BW on leg strength, indicated by the birds’ ability to stand in the LTL test, were only apparent when there were large differences in BW, since CONV birds performed similarly to FAST and MOD birds.

#### Effect of Sex

Males presented poorer leg strength than females as demonstrated by their shorter time standing in the LTL test, greater percentage of birds lying down in the water, and more times lying in the water. These results are corroborated by other studies that indicate poorer walking ability and welfare in males (Sanotra et al., 2001 Dixon, 2020), most likely because males have a faster growth rate and greater BW than females (Dixon, 2020).

### Group Obstacle Test

#### Effect of Category at each TW

The group obstacle test assesses birds’ mobility by determining their ability to cross an obstacle to obtain access to food or water. For lame birds, both standing and moving may be associated with discomfort and or pain (Weeks et al., 2000 Hothersall et al., 2016). Although no significant difference in latency to cross the obstacle was observed among the categories at both TWs, SLOW birds had the lowest BW and greater number of obstacle crossings, suggesting that the differences in BW influenced the ability to cross the obstacle among the categories, but not the initial motivation to cross. In fact, differences in total obstacle crossings were only observed between SLOW and the other categories, despite differences in BW and growth rate among categories.

Because SLOW birds were lighter than the remaining categories and also had the slowest growth rate, it is unknown to which extent the differences in BW and genetic potential for growth caused the differences in total obstacle crossings between SLOW and other categories. While the number of total obstacle crossings at TW 2 appeared to be linearly related to growth rate, CONV, FAST, and MOD did not significantly differ from each other, while SLOW was greater than CONV and FAST birds. Interestingly, despite the lighter BW of CONV birds compared to FAST birds at both TWs, CONV and FAST birds did not differ in total number of obstacle crossings. The lower BW of CONV birds vs*.* FAST may be attributed to the age the group obstacle test was conducted prior to each target weight, particularly in CONV birds, in order to accommodate other behavioral tests and activities performed throughout the study. The results obtained in the group obstacle test partially complement the observations obtained in the LTL test, supporting the hypothesis that selection for accelerated and early muscle accretion may have negative effects on mobility in broiler chickens. The lower number of crossings observed in FG birds may be due to their body conformation (including large breast muscles: Santos et al., 2021a; and short legs: Santos et al., 2021b) requiring an altered gait to increase their stability while walking (Paxton et al., 2014) Corr et al. (2003b). suggested that the gait of FG birds is inefficient, rapidly tiring the birds and leading to low activity levels. The altered gait seen in FG birds may be a result of pain (Corr et al., 1998 McGeown, 1999; Caplen et al., 2013), biomechanical issues related to body conformation, or both (Corr et al., 2003b). The substantial increase in breast muscle observed in FG birds has moved the center of gravity cranially (Corr et al., 2003a, b Paxton et al., 2014). In fact, it has been demonstrated that the center of gravity of FG birds shifts from caudodorsal to craniodorsal between 28 and 42 d, likely as a result of the substantial breast muscle growth in this period, resulting in an increase in muscle forces required to balance it. However, in giant junglefowl, a wild progenitor population of modern chickens, the center of gravity was only reported to move caudodorsally across ontogeny (Paxton et al., 2010, 2014). This change in body conformation observed in FG birds, leading to alterations in forces involved while walking (Corr et al., 1998) and the rapid muscle accretion on the immature skeleton are known to affect locomotion (Corr et al., 2003b). Despite the differences found in the LTL and group obstacle tests between CONV and SLOW birds, CONV birds had similar or greater tibial breaking strength and ash content compared to SG birds (Santos et al., 2021b). Therefore, it seems likely that the differences observed in both mobility tests were not influenced by differences in bone traits among categories.

There are other plausible reasons why categories may have differed in the number of obstacle crossings. Differences in the obstacle test may reflect differences in feeding strategies. Strains selected for differences in feed intake may have differences in feeding behavior related to feeder visits and meal sizes (Barbato et al., 1980 Howie et al., 2009). As part of this larger research project, Dawson et al. (2021) found that while CONV birds spent more time eating, no difference in feeding or drinking bouts was found between the CONV and SG birds. Although the number of visits to the feeder was not evaluated, these findings suggest that the categories and strains evaluated in our study organized their feeding similarly Weeks et al. (2000). reported significant changes in feeding behavior in lame birds, with a decrease in visits to the feeder but increased duration of feeding per bout compared to sound birds, resulting in a similar total time spent feeding per day, despite the differences in feeding strategies. Nevertheless, it is unclear if the differences in total obstacle crossings between SLOW and other categories in our study occurred in response to lameness. Response to the group obstacle test may also be influenced by temperament, motivation to walk and cross the obstacle, behavior and feeding strategies. In fact, a previous study evaluating the same strains reported here, found that differences in growth rate related to differences in inactivity and behavior (e.g., sitting, standing, and walking) among categories (Dawson et al., 2021). Therefore, it is possible that such differences may have influenced leg heath and consequently the results obtained in the group obstacle test LLT test.

As expected, from TW 1 to TW 2, there was a decrease in the number of obstacle crossing in all categories, which may be attributed to the well-known decrease in locomotion with increasing age and BW (Kestin et al., 2001 Dixon, 2020).

#### Differences Among Categories at a Similar Age

The group obstacle test was performed at approximately 41 d for all categories. The increase in BW among categories was accompanied by a significant decrease in total obstacle crossings, with CONV birds crossing fewer times than the remaining categories and FAST and MOD birds being similar but crossing fewer times than SLOW birds.

The CONV birds had a similar latency to cross the obstacle compared to MOD birds, but greater than FAST and SLOW birds. Because CONV birds were heavier and had the greatest feed intake at d 48 (Torrey et al., 2021), it is expected that CONV birds would be equally or more motivated to eat than other birds at the same age, suggesting that a greater latency to cross the obstacle was associated with lack of ability to cross rather than lack of motivation to obtain access to the feeder. However, because MOD birds showed a latency to cross that did not differ from the other categories despite the differences in BW and total obstacle crossings at a similar age, differences in motivation could also contribute to the latency to cross the obstacle. In a study conducted to determine motivation and physical ability to walk for a food reward in FG and SG strains, Bokkers and Koene (2004) revealed that, following a similar feed deprivation period (likely leading to a similar motivation to access the food), SG birds had a shorter latency to start to walk and walked faster down a runway compared to FG birds. The authors concluded that while motivation is the determining factor for walking in lighter birds, physical ability plays a major role in the walking ability of heavy birds.

#### Effect of Sex

Although the increase in BW across categories was associated with fewer obstacle crossings, males were heavier yet crossed the obstacle more often than females, likely due to the higher feed intake of males (Marks, 1985 Benyi et al., 2015) and possibly differences in feeding strategies. Therefore, it is possible that other than differences in mobility, differences in feed motivation may influence the total number of obstacle crossings among sexes, strains, and categories.

### Contact Dermatitis and Litter Moisture

#### Effect of Category at Each TW

Litter quality is the main contributing factor for the occurrence and severity of FPD, with sudden deterioration of litter condition and prolonged contact with poor quality litter increasing the susceptibility of skin lesions (Allain et al., 2009 Shepherd and Fairchild, 2010). To support their rapid growth, FG strains had higher feed intake than SG strains (Torrey et al., 2021), which would result in a higher amount of excreta over a shorter period of time. This most likely accelerated the increase in litter moisture in CONV birds’ pens. At 14 and 28 d, CONV had higher litter moisture than SG pens. However, a similar litter moisture content was observed at 42 d, which was within 1 wk before CONV birds reached TW 2.

The similar incidence of FPD between CONV and SLOW birds observed at TW 1 was unexpected, as a lower incidence of FPD has been reported in SG strains in other studies (Kjaer et al., 2006 Allain et al., 2009; Dixon, 2020). However, CONV had a greater incidence of FPD compared to FAST and MOD birds at TW 1. Litter moisture content measured the week prior to TW 1 for CONV (28 d) and SG categories (42 d) was similar, suggesting that the higher incidence of FPD in CONV birds compared to FAST and MOD birds cannot be solely attributed to differences in absolute values of litter moisture on a pen basis. The SLOW birds had the lowest ADG and average daily feed intake amongst the categories (Torrey et al., 2021). Therefore, drier litter and lower incidences of FPD were expected. However, SLOW birds were observed perching on the waterline, which may have contributed to higher moisture content close to the drinking area. Because samples from different locations of the pens were pooled, the differences in moisture content in distinct areas of the pen between categories were not determined.

The higher incidence of FPD in CONV birds at TW 2 is in line with earlier studies (Kjaer et al., 2006 Allain et al., 2009; Dixon, 2020). Even though BW has not been shown to affect FPD (Kjaer et al., 2006), the higher susceptibility of FG strains to develop FPD may relate to skin integrity (Kafri et al., 1984 Kjaer et al., 2006) or to the heavy BW combined with the lower activity of FG birds that may increase the pressure on the footpads, hocks, and breast, increasing the occurrence of contact dermatitis (Mayne, 2005).

The high incidence of FPD found in CONV birds in our study should be interpreted cautiously due to the high litter moisture content observed throughout the trials. In our study, litter moisture exceeded 30% from 28 d onward for all categories. Litter moisture is a combination of water from spillage, condensation, and excretion (Collett, 2012). Although the amount of excreta produced likely differed among categories due to differences in feed intake, the high litter moisture observed is an indicator of suboptimal environmental conditions. While the solid walls between the pens in the current study prevented visual contact of adjacent pens, this design hindered air circulation at the pen level. It is possible that the standardized housing conditions in our study may have affected FG birds to a greater extent compared to SG birds due to their fast growth and greater amount of excreta produced at an earlier age compared to the other categories (Collett, 2012).

Despite the differences in the total incidence of FPD at TW 1, no difference in the severity of FPD was observed among categories. However, as previously mentioned, CONV birds had the lightest BW at TW 1. Therefore, differences observed at TW 1 may not accurately represent the influences of selection for growth on litter conditions and FPD. At TW 2, CONV had a greater incidence of severe FPD than FAST but was similar to MOD and SLOW. The reason for this difference is unclear.

The incidence of HB was greater in CONV and MOD compared to SLOW birds at TW 1. However, CONV and MOD did not differ from FAST birds. This result was not expected as the increase in BW is commonly associated with a greater incidence of HB (Kjaer et al., 2006), which occurs as a result of the prolonged time sitting and decrease in the locomotor activity commonly observed as the BW increases (Bokkers and Koene, 2003). The lighter BW of CONV birds at TW 1 may explain the lack of difference between CONV, FAST and MOD at the time of the evaluation at TW 1. However, an effect of BW on the incidence of HB was observed at TW 2, when CONV and FAST were heavier at processing (Torrey et al., 2021) and also had greater incidence of HB compared to MOD and SLOW birds.

Overall, the incidence of severe HB was low at both TWs with no significant difference observed among the categories, a finding that differs from other studies (Kjaer et al., 2006 Dixon, 2020; Rayner et al., 2020). Possible explanations for these differences are the different scoring systems used (Allain et al., 2009 Dixon, 2020) or the conditions in which the birds were reared (Dixon, 2020).

#### Differences Among Categories at a Similar Age

At d 48, CONV birds only differed from FAST in incidence and severity of FPD, despite similar litter moisture content among categories at 42 d, suggesting that factors other than absolute litter moisture may have influenced FPD at a similar age. At a similar age, CONV birds had a greater incidence of HB than SLOW birds, while no significant differences were found between CONV, FAST, and MOD birds. The differences between CONV and SLOW birds are congruent with other studies in which a greater incidence and severity of HB was observed in FG compared to SG birds (Bokkers and Koene, 2003 Dixon, 2020; Rayner et al., 2020). However, similar to the results obtained in the LTL test at a similar age, the differences in HB observed in the current study suggest that the negative effects of BW on HB at a similar age may be more obvious when comparing strains greatly differing in growth rate and BW.

#### Effect of Sex

The incidence of HB, which relates to both BW and time spent sitting, was greater in males than females. However, females had a greater incidence of FPD than males. Although the relationship between FPD and sex is unclear, the greater incidence of FPD in females has been attributed to the differences in skin integrity between females and males. (Kamyab, 2001 Mayne, 2005).

### Correlations Analyses

Overall weak positive or negative correlations were observed between the severity of contact dermatitis and the outcome variables obtained in the LTL and group obstacle test. However, because these correlations were weak and inconsistent, contact dermatitis was most likely not the determining factor in differences observed among categories in the mobility tests. These findings agree with Berg and Sanotra (2003) and Ruiz-Feria et al. (2014), who found no correlation between the incidence FPD and LTL test.

Although both LTL and group obstacle tests have been associated with gait scores, they are assessing different aspects of mobility. Even though there were moderate to strong correlations in the variables measured in each test, no correlation between the LTL and group obstacle tests was found for any category. While LTL test assesses birds’ leg strength and ability and/or willingness to stand to avoid lying down in shallow water, the group obstacle test measures birds’ ability to cross the obstacle (i.e., step on and off of the obstacle) and walk to obtain access to resources (food or water) placed on opposite sides of the pen. Therefore, both tests could be used as alternatives to the gait score assessment as suggested by Caplen et al. (2014), but they might indicate different factors associated with walking ability.

## CONCLUSIONS

The effect of both growth rate and BW were observed in most of the variables investigated in this study. While at TW 1, the greater latency to lie down was associated with lower BW, total obstacle crossings per bird were affected by both BW and growth rate. However, at TW 2, growth rate, BW, and likely body conformation (i.e., larger breasts and shorter legs) affected the LTL and total obstacle crossings, with faster-growing birds showing indicators of poorer leg strength and mobility compared to slower-growing birds. These results suggest that as the BW increases, the effects of growth rate on leg strength and mobility become more evident. Most of the variables investigated differed among categories at a similar age and between sexes. Overall, these differences were associated with differences in BW. Although some differences in contact dermatitis suggest the effects of selection for growth, most of the results indicate the relevance of good litter quality and moisture control as effective strategies to decrease the incidence of contact dermatitis in both FG and SG birds. Nevertheless, the incidence and severity of contact dermatitis most likely did not play a major role in the differences observed in the LTL and group obstacle tests, suggesting the differences in indicators of leg strength and mobility obtained in the study were mainly affected by differences in BW, selection emphasis for growth, and likely body conformation.

## ACKNOWLEDGMENTS

This project was possible due to the financial support from Global Animal Partnership and the Canada First Research Excellence Fund (as part of the Food from Thought). In-kind support was received from Ontario Agri-Food Innovation Alliance, the anonymous breeding companies and Protekta, Inc. This project could not have happened without the diligent help of our student assistants (in alphabetical order): Alan Abdulkadar, Breanna Jackson, Erin Ross, Kayley Teal, King (Stella) Hung, Jessica Woods, Leah Wellard, Madeleine Browne, Margaret Daoust, Megan Weckwerth, Melanie Felker, Narissa Leslie, Nyasha Mombeshora, Priyanka Thavaraja, Quinn Rausch, Siobhan Mellors, and Veronica Cheng. Finally, the assistance of the Arkell Poultry Research Station staff was pivotal to this project's success. We thank Dave Vandenberg, Innes Wilson, Nancy Wedel, Vern Wideman, Heidi Naumann, Rick Hoiting, and Danielle Watson for all their help.

## DISCLOSURES

All the authors reviewed the manuscript and approved the submission to Poultry Science and confirmed that the manuscript has not been published or it is under consideration and review by another journal. This project was financially supported by Global Animal Partnership and the Canada First Research Excellence Fund. In-kind support was received from Ontario Agri-Food Innovation Alliance and anonymous breeding companies. We declare no conflict of interest on the publication of this manuscript.

## Appendix Supplementary materials

Image, application 1
