# Supplementary material for: In pursuit of a better broiler: walking ability and incidence of contact dermatitis in conventional and slower growing strains of broiler chickens
Source: Poult Sci. 2022 Jan 31;101(4):101768. doi: 10.1016/j.psj.2022.101768 (PMC8892009; doi:10.1016/j.psj.2022.101768)
Supplement: Supplementary file 1 [file mmc1.docx]

## Effect of Strain on Latency-to-Lie and Group Obstacle Test at Target Weight 1 and 2.

Supplementary Table 1. Differences in body weight (BW), latency-to-lie (LTL), and group obstacle tests (LS means ± SEM) among CONV strains in the week prior to Target Weights 1 and 2. At Target Weight 1 and 2, CONV birds were 34 and 48 days, respectively.

|  | **Strain** | |
| --- | --- | --- |
| **Variable** | **B** | **C** |
| **Target Weight 1** |  |  |
| BW (g)- LTL test^1^ | 1,726 ± 79.6 | 1,735 ± 81.3 |
| LTL (s) | 549.2 ± 39.75 | 460.9 ± 42.07 |
| % of birds lying per pen | 17.1 ± 9.45 | 35.5 ± 11.34 |
| Lying events per bird | 0.42 ± 0.155 | 0.77 ± 0.215 |
| BW (g)- obstacle test^2^ | 1,820 ± 73.8 | 1,764 ± 68.5 |
| No. of obstacle crossings^3^ | 7.14 ± 1.005 | 8.93 ± 0.981 |
| Latency to cross (s) | 898.8 ± 294.21 | 570.4 ± 128.46 |
| **Target Weight 2** |  |  |
| BW (g)- LTL test | 2,992 ± 88.1 | 2,733 ± 85.3 |
| LTL (s) | 340.6 ± 45.76 | 379.8 ± 44.08 |
| % of birds lying per pen | 58.2 ± 10.83 | 60.1 ± 11.88 |
| Lying events per bird | 1.04 ± 0.326 | 1.62 ± 0.421 |
| BW (g)- obstacle test | 2,794 ± 73.8 | 2,485 ± 66.4 |
| No. of obstacle crossings | 4.60 ± 1.002 | 5.85 ± 0.976 |
| Latency to cross (s) | 1,896 ± 609.4 | 2,407 ± 606.1 |

^1^ BW from focal birds tested in the latency-to- lie test. Birds were weighed on the same day the test was conducted.

^2^ BW from focal birds tested in the group obstacle test. Birds were weighed on the same day the test was conducted.

^3^ Number of obstacle crossings per focal bird.

**Supplementary Table 2.** Differences in body weight (BW), latency-to-lie (LTL), and group obstacle test (LS means ± SEM) among FAST strains in the week prior to Target Weights 1 and 2. At Target Weight 1 and 2, FAST birds were 48 and 62 days, respectively.

|  | **Strain** | | | |  |
| --- | --- | --- | --- | --- | --- |
| **Variable** | **F** | **G** | **I** | **M** |  |
| **Target Weight 1** |  |  |  |  |  |
| BW (g)- LTL test^1^ | 2,357 ± 79.5^a^ | 2,442 ± 99.7^a^ | 2,413 ± 99.0^a^ | 1,911 ± 85.5^b^ |  |
| LTL (s) | 425.2 ± 40.18 | 403.7 ± 47.03 | 342.6 ± 46.17 | 403.8 ± 46.03 |  |
| % of birds lying per pen | 51.1 ± 10.73 | 56.9 ± 11.08 | 75.0 ± 11.08 | 48.0 ± 10.66 |  |
| Lying events per bird | 1.96 ± 0.447 | 1.03 ± 0.336 | 1.09 ± 0.342 | 0.62 ± 0.229 |  |
| BW (g)- obstacle test^2^ | 1,967 ± 76.5 | 1,885 ± 79.3 | 1,906 ± 79.3 | 2,253 ± 97.1 |  |
| No. of obstacle crossings^3^ | 5.89 ± 1.044 | 7.14 ± 1.053 | 8.81 ± 1.053 | 9.21 ± 1.290 |  |
| Latency to cross (s) | 1,494 ± 607.5 | 930.8 ± 248.37 | 1,040 ± 296.7 | 799.4 ± 301.27 |  |
| **Target Weight 2** |  |  |  |  | |
| BW (g)- LTL test | 3,485 ± 84.1 | 3,396 ± 99.0 | 3,339 ± 99.0 | 3,109 ± 104.9 | |
| LTL (s) | 404.7 ± 42.83 | 372.5 ± 46.17 | 357.8 ± 46.17 | 410.7 ± 55.97 | |
| % of birds lying per pen | 56.3 ± 11.82 | 58.3 ± 11.08 | 58.3 ± 11.08 | 50.6 ± 13.21 | |
| Lying events per bird | 1.47 ± 0.369 | 1.32 ± 0.397 | 1.42 ± 0.420 | 0.73 ± 0.304 | |
| BW (g)- obstacle test | 3,154 ± 82.2 | 2,990 ± 79.3 | 2,968 ± 79.3 | 2,964 ± 97.1 | |
| No. of obstacle crossings | 4.72 ± 1.122 | 5.56 ± 1.053 | 6.78 ± 1.053 | 8.54 ± 1.290 | |
| Latency to cross (s) | 2,231 ± 751.8 | 2,062 ± 523.1 | 920.4 ± 278.27 | 1,124 ± 376.3 | |

^1^ BW from focal birds tested in the LTL test. Birds were weighed on the same day the test was conducted.

^2^ BW from focal birds tested in the group obstacle test. Birds were weighed on the same day the test was conducted.

^3^ Number of obstacle crossings per focal bird.

^a,b^ Different superscripts within the same row represent differences among categories (P < 0.05).

**Supplementary Table 3.** Differences in body weight (BW), latency-to-lie (LTL), and group obstacle test (LS means ± SEM) among MOD strains in the week prior to Target Weights 1 and 2. At Target Weight 1 and 2, MOD birds were 48 and 62 days, respectively.

|  | **Strain** | | | |
| --- | --- | --- | --- | --- |
| **Variable** | **E** | **H** | **O** | **S** |
| **Target Weight 1** |  |  |  |  |
| BW (g)- LTL test^1^ | 2,220 ± 79.6^a^ | 1,735 ± 72.1^b^ | 2,398 ± 99.0^a^ | 2,208 ± 99.0^a^ |
| LTL (s) | 328.6 ± 39.75 | 486.1 ± 36.46 | 435.2 ± 46.17 | 414.0 ± 46.17 |
| % of birds lying per pen | 64.5 ± 9.45 | 36.8 ± 9.88 | 50.0 ± 11.08 | 50.0 ± 11.08 |
| Lying events per bird | 0.87 ± 0.247 | 0.62 ± 0.717 | 0.96 ± 0.313 | 0.69 ± 0.249 |
| BW (g)- obstacle test^2^ | 2,160 ± 77.2^a^ | 1,821 ± 72.9^ab^ | 1,868 ± 77.2^ab^ | 1,735 ± 77.2^b^ |
| No. of obstacle crossings^3^ | 8.75 ± 1.278 | 8.91 ± 1.267 | 7.11 ± 1.278 | 9.88 ± 1.278 |
| Latency to cross (s) | 1,396 ± 471.9 | 964.3 ± 248.18 | 1,717 ± 416.2 | 909.8 ± 242.22 |
| **Target Weight 2** |  |  |  |  |
| BW (g)- LTL test | 3,350 ± 88.1^a^ | 2,884 ± 73.9^b^ | 3,386 ± 99.0^a^ | 3,098 ± 99.1^ab^ |
| LTL (s) | 397.1 ± 45.76 | 489.8 ± 41.95 | 360.5 ± 46.17 | 449.2 ± 46.20 |
| % of birds lying per pen | 61.7 ± 10.83 | 29.2 ± 11.04 | 58.3 ± 11.08 | 41.7 ± 11.08 |
| Lying events per bird | 0.79 ± 0.267 | 0.57 ± 0.186 | 0.69 ± 0.250 | 0.71 ± 0.257 |
| BW (g)- obstacle test | 2,916 ± 77.2 | 2,683 ± 72.9 | 2,893 ± 77.2 | 2,694 ± 77.5 |
| No. of obstacle crossings | 9.64 ± 1.278 | 6.94 ± 1.279 | 5.67 ± 1.278 | 6.48 ± 1.278 |
| Latency to cross (s) | 2,567 ± 800.8 | 1,337 ± 523.1 | 1,955 ± 730.3 | 1,240 ± 528.0 |

^1^ BW from focal birds tested in the LTL test. Birds were weighed on the same day the test was conducted.

^2^ BW from focal birds tested in the group obstacle test. Birds were weighed on the same day the test was conducted.

^3^ Number of obstacle crossings per focal bird.

^a,b^ Different superscripts within the same row represent differences among categories (P < 0.05).

**Supplementary Table 4.** Differences in body weight (BW), latency-to-lie (LTL), and group obstacle test (LS means ± SEM) among SLOW strains in the week prior to Target Weights 1 and 2. At Target Weight 1 and 2, SLOW birds were 48 and 62 days, respectively.

|  | **Strain** | | | |  |
| --- | --- | --- | --- | --- | --- |
| **Variable** | **D** | **J** | **K** | **N** |  |
| **Target Weight 1** |  |  |  |  |  |
| BW (g)- LTL test^1^ | 1,671 ± 67.1 | 2,082 ± 102.3 | 1,935 ± 99.0 | 1,936 ± 99.0 |  |
| LTL (s) | 460.6 ± 34.68 | 493.5 ± 49.83 | 481.8 ± 46.17 | 516.8 ± 46.17 |  |
| % of birds lying per pen | 36.1 ± 9.22 | 54.2 ± 11.08 | 29.2 ± 11.08 | 20.8 ± 11.08 |  |
| Lying events per bird | 0.51 ± 0.138 | 0.79 ± 0.292 | 0.46 ± 0.189 | 0.46 ± 0.190 |  |
| BW (g)- obstacle test^2^ | 1,684 ± 64.6 | 1,646 ± 71.5 | 1,494 ± 71.5 | 1,516 ± 71.5 |  |
| No. of obstacle crossings^3^ | 11.31 ± 1.332 | 9.92 ± 1.259 | 11.69 ± 1.259 | 11.58 ± 1.259 |  |
| Latency to cross (s) | 856.5 ± 231.68 | 903.3 ± 261.80 | 772.9 ± 406.01 | 1,392 ± 569.7 |  |
| **Target Weight 2** |  |  |  |  | |
| BW (g)- LTL test | 2,797 ± 74.1 | 2,990 ± 99.0 | 2,824 ± 99.0 | 2,603 ± 99.6 | |
| LTL (s) | 514.5 ± 41.82 | 493.3 ± 46.17 | 485.0 ± 46.17 | 435.3 ± 46.96 | |
| % of birds lying per pen | 18.0 ± 11.47 | 37.5 ± 11.08 | 33.3 ± 11.08 | 54.2 ± 11.08 | |
| Lying events per bird | 0.29 ± 0.117 | 0.43 ± 0.182 | 0.48 ± 0.193 | 0.93 ± 0.312 | |
| BW (g)- obstacle test | 2,383 ± 64.6 | 2,545 ± 71.5 | 2,443 ± 78.3 | 2,322 ± 71.5 | |
| No. of obstacle crossings | 10.07 ± 1.248 | 7.44 ± 1.259 | 9.70 ± 1.380 | 9.39 ± 1.259 | |
| Latency to cross (s) | 1,095 ± 257.6 | 819.6 ± 188.69 | 1,059 ± 345.2 | 608.1 ± 205.54 | |

^1^ BW from focal birds tested in the LTL test. Birds were weighed on the same day the test was conducted.

^2^ BW from focal birds tested in the group obstacle test. Birds were weighed on the same day the test was conducted.

^3^ Number of obstacle crossings per focal bird.

## Effect of Category and Sex on Latency-to-Lie Test at Target Weight 1 and 2.

**Supplementary Table 5.** Effect of category and sex and category on body weight (BW) and latency-to-lie (LTL) test (LS means ± SEM) within a week prior Target Weight 1 and Target Weight 2

|  | **Strain** | | | | |  |
| --- | --- | --- | --- | --- | --- | --- |
| **Variable** | **Sex** | **CONV** | **FAST** | **MOD** | **SLOW** |  |
| **Target Weight 1** |  |  |  |  |  |  |
| BW (g)- LTL^1^ | F | 1,619 ± 72.4^bz^ | 2,058 ± 54.1^az^ | 1,933 ± 51.1^az^ | 1,732 ± 53.5^bz^ |  |
|  | M | 1,843 ± 60.0^cy^ | 2,504 ± 50.5^ay^ | 2,347 ± 48.4^ay^ | 2,085 ± 51.0^by^ |  |
| LTL (s) | F | 521.3 ± 45.77 | 453.3 ± 32.13^y^ | 453.3 ± 29.80^y^ | 483.9 ± 30.98 |  |
|  | M | 488.9 ± 33.05^a^ | 334.4 ± 28.60^bz^ | 378.6 ± 26.66^bz^ | 492.4 ± 28.03^a^ |  |
| % of birds lying per pen | F | 25.0 ± 10.49 | 44.5 ± 7.26^z^ | 41.0 ± 6.83^z^ | 36.8 ± 6.95 |  |
|  | M | 27.5 ± 8.50^b^ | 71.0 ± 6.71^ay^ | 59.7 ± 6.41^ay^ | 33.3 ± 6.58^b^ |  |
| **Target Weight 2** |  |  |  |  |  | |
| BW (g)- LTL | F | 2,579 ± 75.8^bcz^ | 2,960 ± 56.5^az^ | 2,785 ± 53.2^bz^ | 2,475 ± 54.1^cz^ | |
|  | M | 3,077 ± 66.1^by^ | 3,704 ± 53.3^ay^ | 3,574 ± 50.0^ay^ | 3,087 ± 51.5^by^ | |
| LTL (s) | F | 426.5 ± 47.04^y^ | 514.3 ± 33.45^y^ | 534.3 ± 31.81^y^ | 532.9 ± 31.71^y^ | |
|  | M | 293.9 ± 38.38^bz^ | 258.5 ± 30.30^bz^ | 314.0 ± 28.68^bz^ | 431.1 ± 28.87^az^ | |
| % of birds lying per pen | F | 44.7 ± 10.77^z^ | 29.1 ± 7.67^z^ | 20.9 ± 7.25^z^ | 26.1 ± 7.27^z^ | |
|  | M | 73.6 ± 9.77^ay^ | 82.6 ± 7.32^ay^ | 74.5 ± 6.81^ay^ | 45.4 ± 6.94^by^ | |

^1^ BW from focal birds tested in the LTL test. Birds were weighed on the same day the test was conducted.

^a-c^ Different superscripts within the same row represent significant differences between categories for each sex (P<0.05).

^y-z^ Different superscripts within the same column for the same parameter represent significant differences between sexes in each category (P<0.05).

## Effect of Strain on Incidence and Severity of Footpad Dermatitis (FPD) and Hock Burns (HB) at Target Weights 1 and 2.

1. B)

**Supplementary Figure 1.** Effects of strains (within category) on total incidence of footpad dermatitis (LS-means ± SEM) at Target Weights 1 (A) and 2 (B). At Target Weight 1, CONV and other categories were 34 and 48 d of age, respectively. At Target Weight 2, CONV and other categories were 48 and 62 days, respectively.

1. B)

**Supplementary Figure 2.** Effects of strains (within category) on total incidence of severe footpad dermatitis (LS-means ± SEM) at Target Weights 1(A) and 2 (B). At Target Weight 1, CONV and other categories were 34 and 48 d of age, respectively. At Target Weight 2, CONV and other categories were 48 and 62 days, respectively.

1. B)

**Supplementary Figure 3.** Effects of strains (within category) on total prevalence of hock burns (LS-means ± SEM) at Target Weights 1 (A) and 2 (B). At Target Weight 1, CONV and other categories were 34 and 48 d of age, respectively. At Target Weight 2, CONV and other categories were 48 and 62 days, respectively. Within category, columns with different superscripts differ (P < 0.05).

1. B)

**Supplementary Figure 4.** Effects of strains (within category) on total incidence of severe scores of hock burns (LS-means ± SEM) at Target Weights 1 (A) and 2 (B). At Target Weight 1, CONV and other categories were 34 and 48 d of age, respectively. At Target Weight 2, CONV and other categories were 48 and 62 days, respectively.

.

## Differences in Litter Moisture among Strains from Day 14 to Day 56.

1. B)

C) D)

**Supplementary Figure 5.** Litter moisture (LS-means ± SEM) among CONV (A), FAST (B), MOD (C) and SLOW (D) birds from 14 to 56 d. Litter moisture content from CONV birds at 56 d is not presented because pens containing CONV birds were processed at 34 and 48 d.


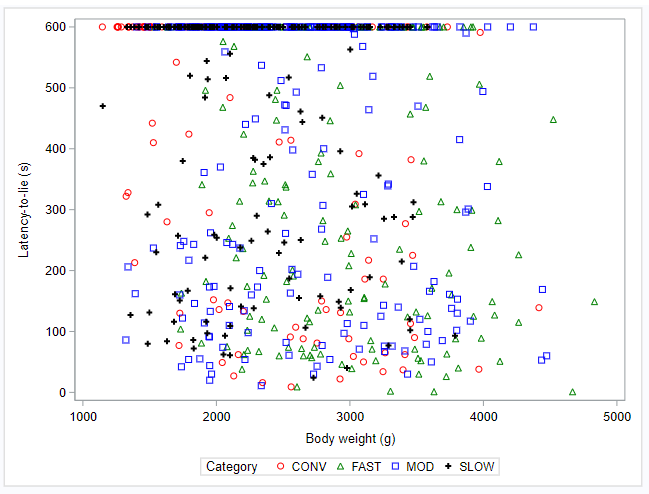


**Supplementary Figure 6.** Scatterplot of latency-to-lie and body weight by category.

***
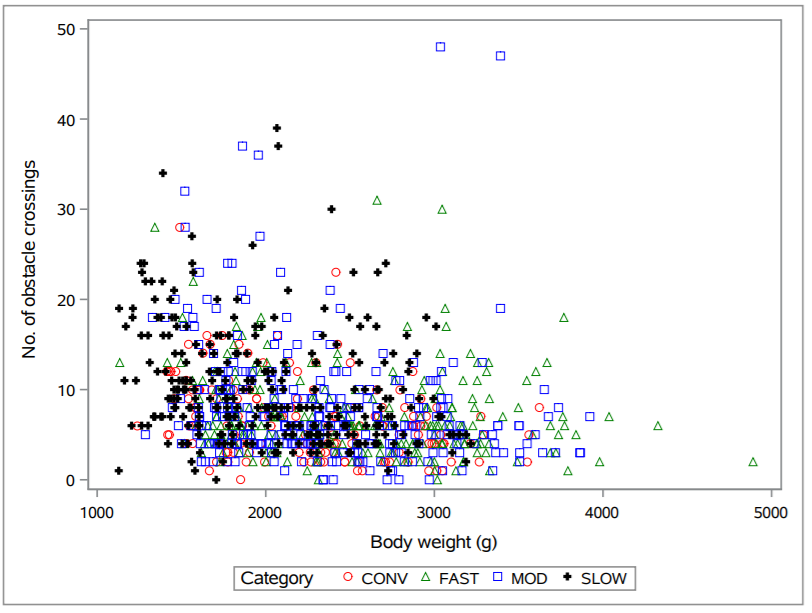
***

**Supplementary Figure 7.** Scatterplot of total obstacle crossings and body weight (BW) by category.

***Effect of strain***

***Latency-to-lie test***. During the LTL test, both CONV strains had an increasing percentage of birds lying down and number of times lying down in the water as well as a decreasing time standing from TW 1 to TW 2. However, this pattern was not observed for some SG strains that showed similar LTL or even a slight increase in LTL from TW 1 to TW 2, suggesting that the increase in BW may not have obvious negative effects on leg strength assessed via LTL test for some strains. Alternatively, this may indicate differences in aversion to water among strains. Furthermore, it should be noted that different birds were tested at each TW. Therefore, individual birds’ variation in aversion to water may have influenced the differences observed in each strain from TW 1 to TW 2.

***Group obstacle test.*** Strains within category did not differ in total obstacle crossings or latency to cross the obstacle, suggesting similar feeding strategies in strains selected for similar growth rates. All strains showed a numeric decrease in total crossings in the obstacle test from TW 1 to TW 2, except strain E (MOD), which had a slight increase. Others have reported a decrease in locomotor activity and walking ability as birds grow, which occurs in both FG and SG strains despite the differences in selection emphasis for growth (Kestin et al., 2001; Bokkers and Koene, 2003).

***Contact dermatitis.*** Few significant or consistent differences were found among strains within categories, which may be due to the large variation or the lack of statistical power, which was lower than 0.70 for most of the variables evaluated, preventing the detection of differences among strains. The two CONV strains differed in HB incidence at TW 1 but not TW 2.

**REFERENCES- Supplementary material**

Bokkers, E. A. M., and P. Koene. 2003. Behaviour of fast- and slow growing broilers to 12 weeks of age and the physical consequences. Appl. Anim. Behav. Sci. 81:59–72.

Kestin, S. C., S. Gordon, G. Su, and P. Sørensen. 2001. Relationships in broiler chickens between lameness, liveweight, growth rate and age. Vet. Rec. 148:195–197.
